# Optical Control of Membrane Viscosity Modulates ER-to-Golgi Trafficking

**DOI:** 10.1021/acscentsci.5c00606

**Published:** 2025-08-12

**Authors:** Noemi Jiménez-Rojo, Suihan Feng, Johannes Morstein, Stefanie D. Pritzl, Antonino Asaro, Sergio López, Yun Xu, Takeshi Harayama, Nynke A. Vepřek, Christopher J. Arp, Martin Reynders, Alexander J. E. Novak, Evgeny Kanshin, Jan Lipfert, Beatrix Ueberheide, Manuel Muñiz, Theobald Lohmüller, Howard Riezman, Dirk Trauner

**Affiliations:** 1 NCCR Chemical Biology, Department of Biochemistry, University of Geneva, CH-1211 Geneva, Switzerland; 2 Department of Biochemistry and Molecular Biology, University of the Basque Country (UPV/EHU), 48940 Leioa, Spain; 3 Ikerbasque, Basque Foundation for Science, 48013 Bilbao, Spain; 4 Unit of Chemical Biology and Lipid Metabolism, Key Laboratory of Immune Response and Immunotherapy, Shanghai Institute of Immunity and Infection, Chinese Academy of Sciences, Shanghai 200031, China; 5 Department of Chemistry, 5894 New York University, New York, New York 10012, United States; 6 Division of Chemistry and Chemical Engineering, 6469 California Institute of Technology, Pasadena, California 91125, United States; 7 Chair of Photonics and Optoelectronics, Nano-Institute Munich, Department of Physics, Ludwig Maximilian University, 80539 Munich, Germany; 8 Soft Condensed Matter and Biophysics, Department of Physics and Debye Institute for Nanomaterials Science, 8125 Utrecht University, 3584 CS Utrecht, The Netherlands; 9 Institute of Bioengineering (IBI) and Global Health Institute, École Polytechnique Fédérale de Lausanne (EPFL), 1015 Lausanne, Switzerland; 10 Department of Cell Biology, Faculty of Biology, University of Seville and Instituto de Biomedicina de Sevilla (IBiS), Hospital Universitario Virgen del Rocío/CSIC/Universidad de Sevilla, 41012 Seville, Spain; 11 Institut de Pharmacologie Moléculaire et Cellulaire, CNRS, Université Côte d’Azur − CNRS UMR1715 − Inserm, U1323 Valbonne, France; 12 Department of Chemistry, Ludwig Maximilian University, 80539 Munich, Germany; 13 NYU Grossman School of Medicine, New York, New York 10016, United States; 14 Institute for Physics, Augsburg University, 86159 Augsburg, Germany; 15 Department of Chemistry, College of Arts and Sciences, University of Pennsylvania, Philadelphia, Pennsylvania 19104-6323, United States

## Abstract

The lipid composition of cellular membranes is highly dynamic and undergoes continuous remodeling, affecting the biophysical properties critical to biological function. Here, we introduce an optical approach to manipulate membrane viscosity based on an exogenous synthetic fatty acid with an azobenzene photoswitch, termed **FAAzo4**. Cells rapidly incorporate **FAAzo4** into phosphatidylcholine and phosphatidylethanolamine in a concentration- and cell type-dependent manner. This generates photoswitchable PC and PE analogs, which are predominantly located in the endoplasmic reticulum. Irradiation causes a rapid photoisomerization that decreases membrane viscosity with high spatiotemporal precision. We use the resulting “PhotoCells” to study the impact of membrane viscosity on ER-to-Golgi transport and demonstrate that this two-step process has distinct membrane viscosity requirements. Our approach provides an unprecedented way of manipulating membrane biophysical properties directly in living cells and opens novel avenues to probe the effects of viscosity in a wide variety of biological processes.

## Graphical abstract

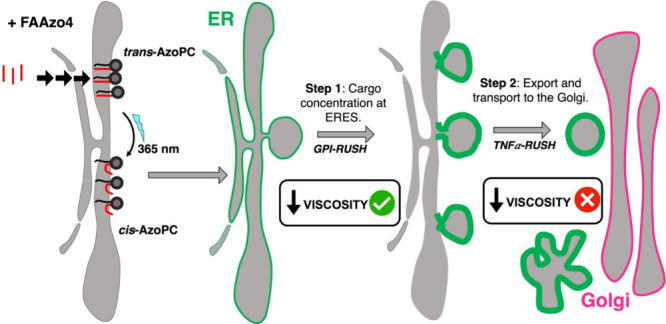

## Introduction

The ability to fine-tune membrane properties is essential to maintaining cellular life.^1^ These properties arise from the complex interplay of thousands of lipids and membrane proteins, which undergo constant remodeling.^2^ Membrane homeostasis relies on complex interdependent processes that are difficult to dissect and study in living systems with standard genetic and biochemical techniques.^3,4^ In fact, controlled manipulations of membrane viscosity have been restricted mostly to *in vitro* studies using model membrane systems, while *in cellulo*, these approaches have been limited to the use of lipid metabolic interventions that modify membrane lipid composition. Treatment of cells with polyunsaturated fatty acids (PUFAs) has been shown to facilitate membrane deformation in the context of endocytosis,^5^ and decreases viscosity and increases permeability to facilitate apoptosis.^6^ Similarly, modifications of lipid saturation levels have been shown to modulate mitochondrial respiration.^7^ These examples emphasize the importance of adjusting membrane viscosity for the proper functioning of physiological processes.

Optogenetics and photopharmacology allow for optical control of biological processes with the spatiotemporal resolution of light. While optogenetics is based on genetically encoded photoreceptors, photopharmacology relies on synthetic molecular photoswitches, such as azobenzenes.^8–10^ Photoswitchable lipids have emerged as versatile tools to control defined protein–membrane interactions *in vivo* ^11,12^ as well as membrane mechanics in model membranes.^13–15^ If photoswitchable lipids could be integrated into cellular membranes in sufficient quantities, they could meet a long-standing desire to control the biophysical parameters of membranes remotely and with high spatiotemporal resolution within living systems.

Here, we engineer the lipid composition of cellular membranes using a synthetic photoswitchable fatty acid that allows us to manipulate the membrane viscosity with light. The synthetic fatty acid **FAAzo4** is efficiently incorporated into glycerophospholipids, mostly phosphatidylcholine (PC) and phosphatidylethanolamine (PE), and integrated in the endoplasmic reticulum (ER) of mammalian cells. Our new methodology enables us to directly modulate membrane viscosity and study the influence of this biophysical parameter on protein secretion.

## Results and Discussion

### Metabolic Engineering of Membrane Lipid Composition Using a Photoswitchable Lipid

To study the capacity of the photoswitchable lipid **FAAzo4** to be taken up by cells and metabolized to give rise to photoswitchable phospholipids (Figure 1A, B), we supplemented growth medium with analogs of **FAAzo4**, incubated HeLa cells for 4 h, and subsequently conducted lipid extraction and mass spectrometric quantification of lipid metabolites. We hypothesized that the cellular uptake of free fatty acids could be limiting for its metabolic incorporation, and therefore, we synthesized and tested a series of esterified pro-**FAAzo4** analogs. Methyl-, ethyl-, and *n*-butyl-esters are common pro-drugs for carboxylic acids^16^ and acetoxymethyl esters are frequently used to mask carboxylic acids in chemical probes.^17^ All of these pro-**FAAzo4** analogs are hydrolyzed intracellularly by nonspecific esterases. While pro-**FAAzo4** (Figure 1B, R^1^ = Me, Et, Bu, AM) analogs exhibited good cell permeability, free **FAAzo4** (R^1^ = H) yielded even higher cellular levels of **AzoPC** (Figure S1A, B).

**Figure 1:**
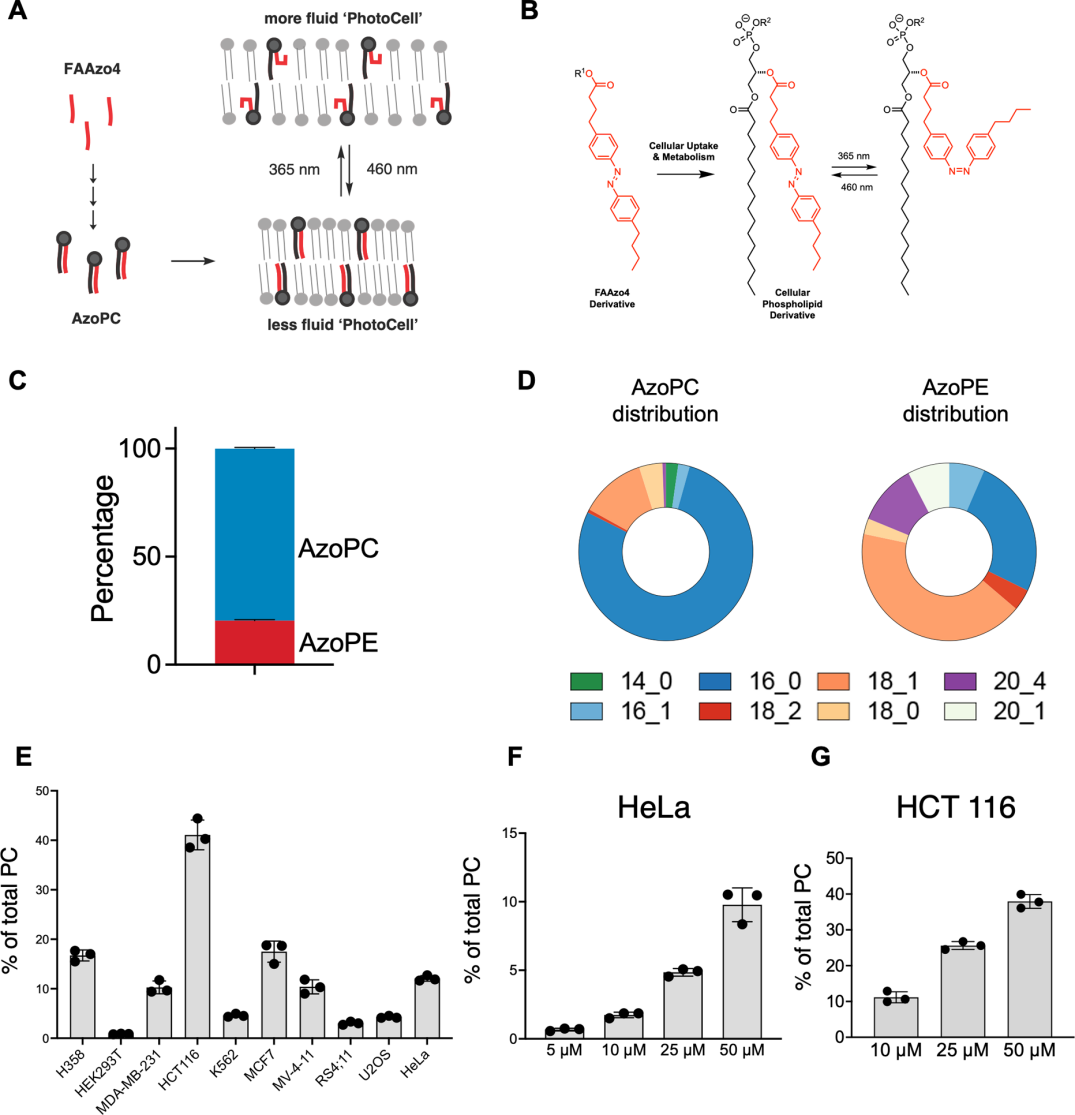
**FAAzo4 is incorporated into glycerophospholipids**. (A, B) Schematic illustration of PhotoCell design and photolipid structures. (C) Proportion of incorporated photolipids after treatment of **FAAzo4** in HeLa cells. (D) Distribution of incorporated photolipids after **FAAzo4** treatment in HeLa cells. (E) Incorporation efficiency of **FAAzo4** in different cell lines. Data represent three biological replicates. (F,G) Concentration dependence of incorporation. HeLa and HCT 116 cells were incubated with **FAAzo4** at indicated concentrations. Data represent three biological replicates. Error bars represent SD, *p < 0.05, **p < 0.01, ***p < 0.001, Student’s *t*-test.

Having settled on **FAAzo4** as the most promising metabolic precursor, we then analyzed the composition of glycerophospholipids containing these synthetic lipids in HeLa cells. Through LC-MS analysis, we found that photoswitchable **AzoPC** and **AzoPE** were the predominant species (Figure 1C, D). Interestingly, we did not detect the incorporation of **FAAzo4** into phosphatidylinositol (PI), phosphatidylserine (PS), and phosphatidylglycerol (PG), indicating selective integration into only the major membrane phospholipids. Notably, incorporation into sphingolipids was also not observed.

To quantify the azobenzene-containing lipid metabolites, we synthesized C17-**AzoPC**, comprised of heptadecanoic acid (C17:0) and **FAAzo4**, and used it as an internal standard. While C17-**AzoPC** is expected to closely mimic the ionization efficiency of **AzoPC**s, we also found that the MS profiles from **AzoPC** and endogenous PC species are similar, suggesting that the headgroup is the decisive factor during the ionization process in our measurement (Figure S1E–G). Accordingly, the amount of **AzoPE** was determined together with endogenous PE species by the same molecular standard (PE31:1). We observed that the predominant form of **AzoPC** was C16:0, which corresponds to the chain length of the most abundant endogenous PC formed and the major **AzoPE**s were C16:0 and C18:1 (Figure 1D). The presence of **AzoPC** and **AzoPE** was further confirmed by LC-MS/MS analysis (Figure S1E, F), in which the cutoff was set at *m*/*z* 200 to bypass the phosphocholine fragment (*m*/*z* 184.07), the major ion from PC species that suppresses other signals. The expected fragments of **FAAzo4** (*m*/*z* 307.18, 325.19) were clearly visible in all **AzoPC**s, **AzoPE**s, as well as in the reference compound C17-**AzoPC** (Figure S1G).

We next explored the mechanisms of **FAAzo4** incorporation using **AzoPC** production quantification as a readout. To determine whether cellular uptake or metabolic incorporation are rate-limiting, we conducted a wash out experiment, in which **FAAzo4** was washed out after 30 min treatment and the incorporation efficiency was measured at several time points up to 4 h after the wash-out. We found that the levels of **AzoPC** did not change after the wash-out indicating that cellular uptake is the rate-limiting step for both **AzoPC** (Figure S1C) and **AzoPE** incorporation (Figure S1D), and that the incorporation likely goes through the phospholipid remodeling pathways.^18^ To test if incorporation is catalyzed by members of the acyl-CoA synthetase long-chain ligase enzymes (ACSLs), we quantified levels of **AzoPC** in ACSL4-KO cells (Figure S1H) and used an ACSL inhibitor triacsin C^19^ (Figure S1I). In both cases we found that the levels of **AzoPC** formed in PhotoCells was reduced compared to the control, indicating the involvement of ACSLs during the incorporation. We also observed that *trans*-**FAAzo4** was incorporated more efficiently than *cis*-**FAAzo4** (Figure S1J), which was obtained through pulsed irradiation with 370 nm light (75 ms every 15 s) using a Cell DISCO system.^20,21^ The amount of incorporated **AzoPC** could be markedly increased through a serum starvation depleting medium of other fatty acids (Figure S1K). Cell viability of PhotoCells was tested in a dose-dependent fashion and compared to the lipotoxic fatty acid palmitic acid (Figure S1L, M). We found that **FAAzo4** treatment did not compromise cell viability, indicating that **AzoPC** was well tolerated in the membranes of living cells.

To test the generality of our PhotoCell approach, we tested FAAzo4 incorporation in a range of adherent and suspension cell lines (Figure 1E). Several cell lines showed effective incorporation, yielding 10–20% AzoPC, including H358, MDA-MB-231, MCF7, MV-4–11, and HeLa cells. Interestingly, HEK293T cells do not exhibit effective incorporation, whereas HCT116 cells showed exceptional incorporation yielding up to 40% AzoPC. To assess the incorporation efficiency of FAAzo4 in HCT116 cells, we treated them with different concentrations of FAAzo4 and found that treatment with 10 μM FAAzo4 in HCT116 cells yielded levels of AzoPC equivalent to treatment with 50 μM FAAzo4 in HeLa cells (Figure 1F, G). Based on these results, we decided to use HeLa and HCT116 PhotoCells in subsequent experiments.

### Remodeled Azo-Phospholipids are Located at the ER Membrane

We next examined the subcellular localization of photolipids in HeLa cells to determine which membranes could be studied with this approach. Both PC and PE are synthesized *de novo* and undergo phospholipid remodeling^18^ in the endoplasmic reticulum although some PE is derived from PS decarboxylation in the mitochondria, which suggests that the ER may contain large amounts of the newly synthesized analogs **AzoPC** and **AzoPE**.^22^ To test if Azo-phospholipids can be detected in the ER, we employed a clickable analogue of **FAAzo4**, termed **clFAAzo4** (Figure 2A). Through minimal chemical modification with a terminal alkyne, Copper-Catalyzed Azide–Alkyne Cycloaddition (CuAAC) can be used on fixed cells to visualize the location of lipid metabolites that underwent PFA fixation (Figure 2B).^23^ **AzoPE** is likely more effectively cross-linked than **AzoPC** due to the free amine occurring on the headgroup making **AzoPE** the predominant species detected. The soluble fluorophore SulfoCy5 is ideally suited for this experiment as its high water solubility prevents accumulation in membranes, enhancing the contrast when it is conjugated to a membrane lipid with two acyl tails.^24,25^ Subsequent addition of an ER marker enabled colocalization. This experiment showed strong overlap of the detected photoswitchable phospholipids with membranes of the ER (Pearson Coefficient of 0.95, Figure 2B), suggesting that PhotoCells could be particularly suited to the study of ER membrane biophysical properties. We also costained with organelle markers for endosomes and the plasma membrane to test if we can detect photoswitchable phospholipids in these compartments with our methods and found no significant colocalization with these organelles (Figure S2). While these experiments suggest predominant localization of photoswitchable phospholipids to the ER, we cannot rule out some localization to other organelles (e.g., Golgi and Mitochondria). Consistent with the absence of photoswitchable phospholipids at the plasma membrane, no meaningful change in the phosphoproteome was detected upon PhotoCell irradiation (Figure S3A, B). We also did not detect a light-dependent effect on vesicular stomatitis virus (VSV) infection, a commonly used model to examine cell entry via endocytosis (Figure S1N, O). This suggests that our system is particularly well suited to studying ER-dependent processes.

**Figure 2:**
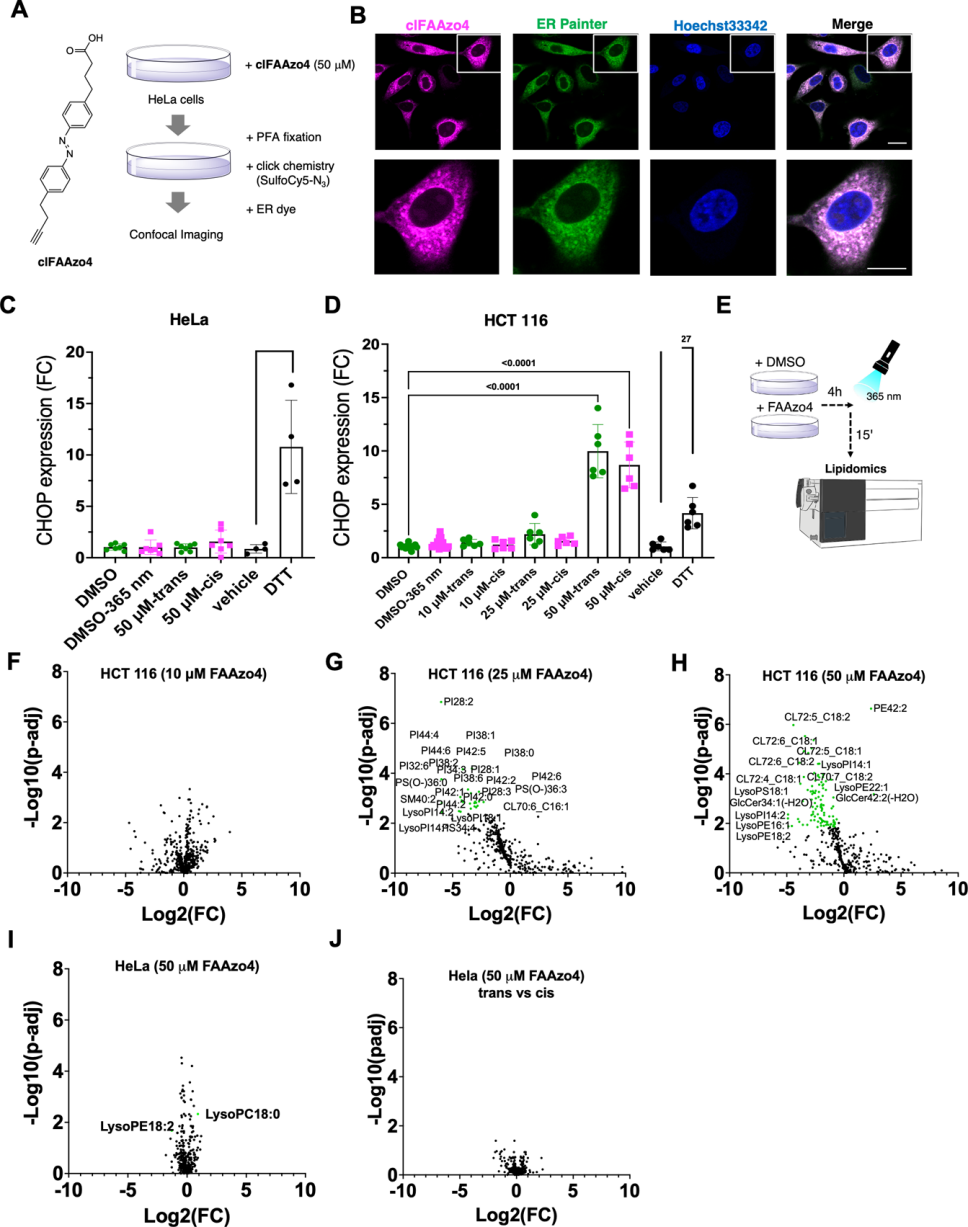
**Incorporation and click-imaging of alkyne-modified FAAzo4 and effect on ER stress and overall lipid composition**. (A) Chemical structure of clickable analog **clFAAzo4** and schematic of click-imaging protocol for incorporated **clFAAzo4** in fixed cells. HTC116 cells were incubated with **clFAAzo4**, fixed with para-formaldehyde, and then labeled with sulfo-cyanin-5-azide by means of CuAAC. Subsequently, cells were treated with a fixation-compatible ER-selective dye. (B) Confocal images (63×) were obtained using λ_Ex_ = 646 nm for Cy5, λ_Ex_ = 561 nm for ER Painter (BODIPY TR Glibenclamide), and λ_Ex_ = 405 nm for Hoechst33342. Scale Bar: 20 μm. (C) Expression of the ER stress marker CHOP upon 4 h of **FAAzo4** treatment in HCT 116 cells before and 15 min after irradiation. (D) Expression of the ER stress marker CHOP upon 50 μM **FAAzo4** treatment in HeLa cells before and after irradiation. (E) Schematic of protocol for cell harvesting for lipidomics analysis of cellular lipid composition. HeLa or HCT 116 cells were incubated with DMSO or **FAAzo4** for 4 h, then irradiated for 2 min and then harvested after 15 min. (F–H) Lipidomic analysis of HCT cells treated with 10, 25, and 50 μM **FAAzo4**. (I) Lipidomic analysis of HeLa cells treated with 50 μM **FAAzo4**. (J) Lipidomic analysis of HeLa cells treated with 50 μM **FAAzo4** after irradiation. Data and statistical analysis were obtained using LipiSig Webtool;^50^ lipids exhibiting significant changes are labeled in the correspondent graphs.

To test if azolipids could perturb ER function, we analyzed RNA levels of C/EBP homologous protein (CHOP), as a readout of ER-stress and consequent activation of the unfolded protein response (UPR). Indeed, we found that levels higher than 25% **AzoPC** led to ER stress and UPR activation (Figure 2C, D). These data were confirmed using the nanostring technology, a multiplex nucleic acid hybridization technology that enables the assessment of the expression of multiple targets, and we found a similar dose-dependent increase of multiple target genes in UPR and SREBP pathways like CHOP, XBP-1, ATF4 or HMGCS1 (Figure S3C–J). Importantly, the photoactivation of **FAAzo4** containing lipids did not have any effect on the expression of those genes. Therefore, the amount of **FAAzo4** incorporation should be carefully monitored and optimized in each biological system to avoid side effects. Cells that have 10% of their cellular PC substituted with **AzoPC**, (i.e., HeLa cells treated with 50 μM **FAAzo4** or HCT 116 cells treated with 10 μM **FAAzo4**) appear to be physiologically unaltered and offer a more relevant background for the study of cellular processes, while cells that have 40% of their PC in the form of **AzoPC** undergo ER stress (Figure 2C, D). This fits well with our observation that 10 μM treatment in HCT 116 cells has little effect on the overall lipidome of the cells, while 25 and 50 μM lead to the formation of several secondary metabolites of cellular phospholipids (Figure 2E, F–H). In agreement with these data, 50 μM **FAAzo4** treatment in HeLa cells or photoactivation of the Azo-lipids did not significantly affect the overall lipidome of these cells (Figure 2I,J).

It is worth mentioning that the formation of secondary phospholipid metabolites at high **FAAzo4** concentrations in HCT116 cells could stem from aberrant lipid trafficking. For instance, when HCT 116 are treated with 25 μM of **FAAzo4**, several PI species are found to be decreased which can be indicative of membrane trafficking defects.^26^ At 50 μM **FAAzo4**, the levels of several cardiolipins are affected which can indicative of secondary effects on mitochondrial function, metabolism and dynamics.^7^

### Photolipids Enable Optical Control of Membrane Viscosity in Model Membranes and Living Cells

To investigate whether **FAAzo4**-containing lipids elicit a change in membrane viscosity upon photoconversion, we performed fluorescence recovery after photobleaching (FRAP) experiments in model membranes and living cells.

We chemically synthesized the predominant cellular metabolite of FAAzo4, C16-AzoPC and performed measurements on supported lipid bilayers (SLBs) as a common model system (Figure 3B–F). We previously reported that photoisomerization of photolipid model membranes containing C18-AzoPC impacts the membrane viscosity.^27^ We observed similar effects in C16-AzoPC containing model membrane systems using ratios of AzoPC:POPC similar to those measured *in cellulo* using lipidomics analysis (1:9) as well as membranes composed of pure C16-AzoPC (Figure 3B–F). In the case of pure C16-AzoPC doped with 1 mol % Texas Red-DHPE for the FRAP measurements, the average diffusion coefficient of the fluorescently labeled lipids in a *trans*-adapted SLB was *D*_*trans*_ = (0.37 ± 0.02) μm^2^ s^–1^ (Figure 3B, C). After the illumination was changed to UV-A light, the diffusion coefficient increased by a factor of ∼4. The average lateral diffusion coefficient of a *cis*-adapted SLB is *D*_*cis*_ = (1.62 ± 0.06) μm^2^ s^–1^, which is indicative for a fluid bilayer membrane.^28^ This increase in diffusion coefficient suggests a decrease in viscosity that can be explained by a decrease of attractive lipid–lipid interactions between photolipids in the *cis* state compared to *trans* molecules, as we showed previously for C18-AzoPC membranes.^27,29^ We further performed temperature dependent FRAP experiments and heated a C16-AzoPC SLB in steps of ∼10 °C up to 60 °C (Figure 3C). We next repeated these experiments with AzoPC:POPC ratios representative of PhotoCells (1:9) (Figure 3E–F). We observed the same trend under these conditions, with an increase of average lateral diffusion coefficient from *D*_*trans*_ = (0.79 ± 0.04) μm^2^ s^–1^ to *D*_*cis*_ = (1.03 ± 0.07) μm^2^ s^–1^ upon *trans*-to-*cis* isomerization (Figure 3E).

**Figure 3:**
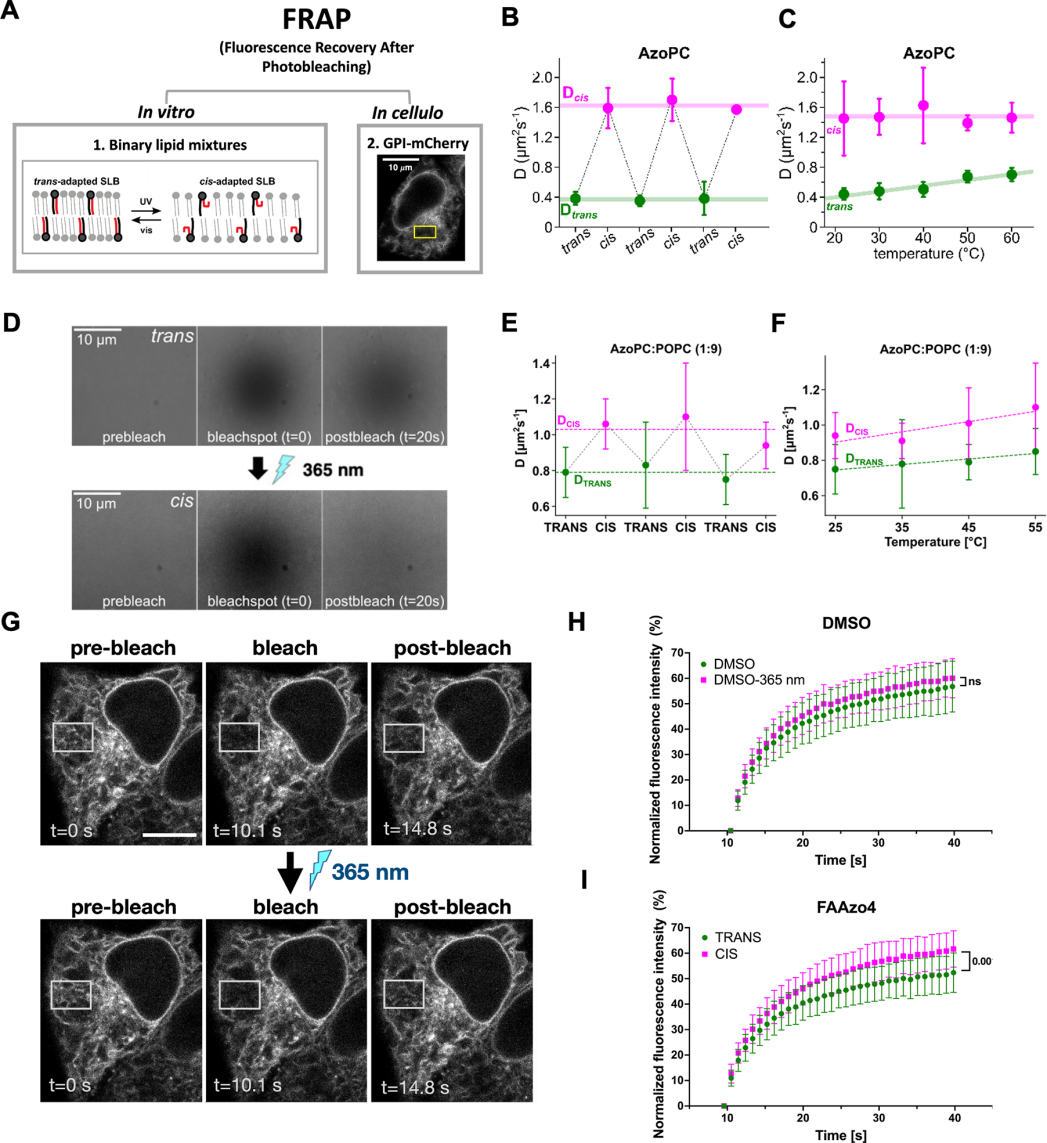
**Fluorescence recovery after photobleaching (FRAP) experiments of Azo-lipid containing membranes in vitro and in cellulo**. (A) Schematic depiction of FRAP experimental approach using model membrane systems (*in vitro*, depiction of light-induced membrane fluidity modulation with **AzoPC**) or living cells (*in cellulo*). (B) Diffusion coefficients of fluorescent lipids in C16-**AzoPC** SLB + 1 mol % Texas Red-DHPE. (C) Temperature dependence of diffusion coefficients of the C16-**AzoPC** SLB + 1 mol % Texas Red-DHPE. (D) Fluorescence images of a SLB, illustrating the bleach spot and fluorescence recovery of a FRAP experiment before and after irradiation. (E) Diffusion coefficients of SLB composed of C16-**AzoPC**:POPC (9:1) + 1 mol % Texas Red-DHPE. (F) Temperature dependence of diffusion coefficients of SLB composed of C16-**AzoPC**:POPC (9:1) + 1 mol % Texas Red-DHPE. (G) Representative images of a FRAP experiment in HeLa cells transfected with va GPI-mCherry construct hooked to the ER. Scale Bar: 10 μm. (H) FRAP kinetics of GPI-mCherry construct in HeLa cells treated with DMSO before and after irradiation. (I) FRAP kinetics of GPI-mCherry construct in HeLa cells treated with FAAZo4 before and after irradiation.

To test whether our system enables optical modulation of membrane viscosity in living cells, we expressed GPI-mCherry in PhotoCells (HeLa cells fed with FAAzo4) and performed FRAP experiments following the protein diffusion at the ER membrane at 37 °C. Photoswitching was controlled using a fluorescence microscope equipped with a 365 nm UV lamp, and FRAP measurements were performed on the same regions of interest (ROI) (Figure 3G). As shown in Figure 3H, after the photoconversion of FAAzo4 from *trans* to *cis*, the fluorescence recovery was faster, as represented by the half-time of the recovery (Figure 3I). In cells treated with DMSO, UV light illumination had no significant effect on protein diffusion kinetics (Figure S4E). Moreover, the extent of the recovery represented by the plateau (Figures 3H and S4C) was also higher after photoconversion, suggesting less immobile fraction of the protein construct when membrane viscosity is lower. This confirms that the rapid isomerization of FAAzo4-containing phospholipids from *trans* to *cis* in living cells decreases the ER membrane viscosity. As a control, we also performed FRAP measurements on SLBs that were made of lipids extracted from PhotoCells and found an increase in diffusion coefficient and immobile fractions upon *trans*-to-*cis* switching which is in excellent agreement with the observed *in-cellulo* viscosity change (Figure S4F).

### ER Membrane Viscosity Controls ER-to-Golgi Transport

One of the major functions of the ER is the export of newly synthesized proteins by the COPII machinery.^30^ This process involves accumulation of the cargo at ER exit sites (ERES), recruitment of the COPII components to the ER membrane, and membrane bending and fission events that give rise to the carriers that transport proteins from the ER to the Golgi. This operation has been previously shown to be modulated by lipid composition, which affects both lipid–protein interactions and membrane mechanical properties necessary for bending and fission.^31–34^ Membrane tension has also been proposed to play a role in carrier exit from the ER especially when large cargoes such as collagens need to be transported.^35^ Thus, we tested whether membrane viscosity plays a role in protein export from the ER. For this purpose we took advantage of the Retention Using Selective Hooks (RUSH) system, an approach to synchronize and follow the transport of proteins^36^ and optimized it both in HeLa and HCT 116 cells (Figure S5A). Combining our approach, which allows for optical control of membrane viscosity, with the RUSH assay, we were able to evaluate the contribution of membrane viscosity to the export of mCherry-TNFα (TNFα-RUSH) and a model EGFP-GPI-anchored protein (GPI-RUSH) (Figure 4). We chose these two constructs, because they have been shown to have distinct localizations and export dynamics. In this context, before biotin addition (time = 0 min) the GPI-RUSH protein is homogeneously dispersed through the ER, while TNFα-RUSH is located in discrete regions that correspond to ERES (Figure S5B), as previously described by Weigel et al.^37^ The prelocalization of TNFα-RUSH to ERES is also supported by the fact that the arrival of TNFα-RUSH to the Golgi started very soon after its release with biotin (Figure 4H). Therefore, transport kinetics of the export of the two different cargos is likely representative of the two stages of the process: (1) recruitment from ER periphery to ERES; and (2) export from ERES to the Golgi.

**Figure 4:**
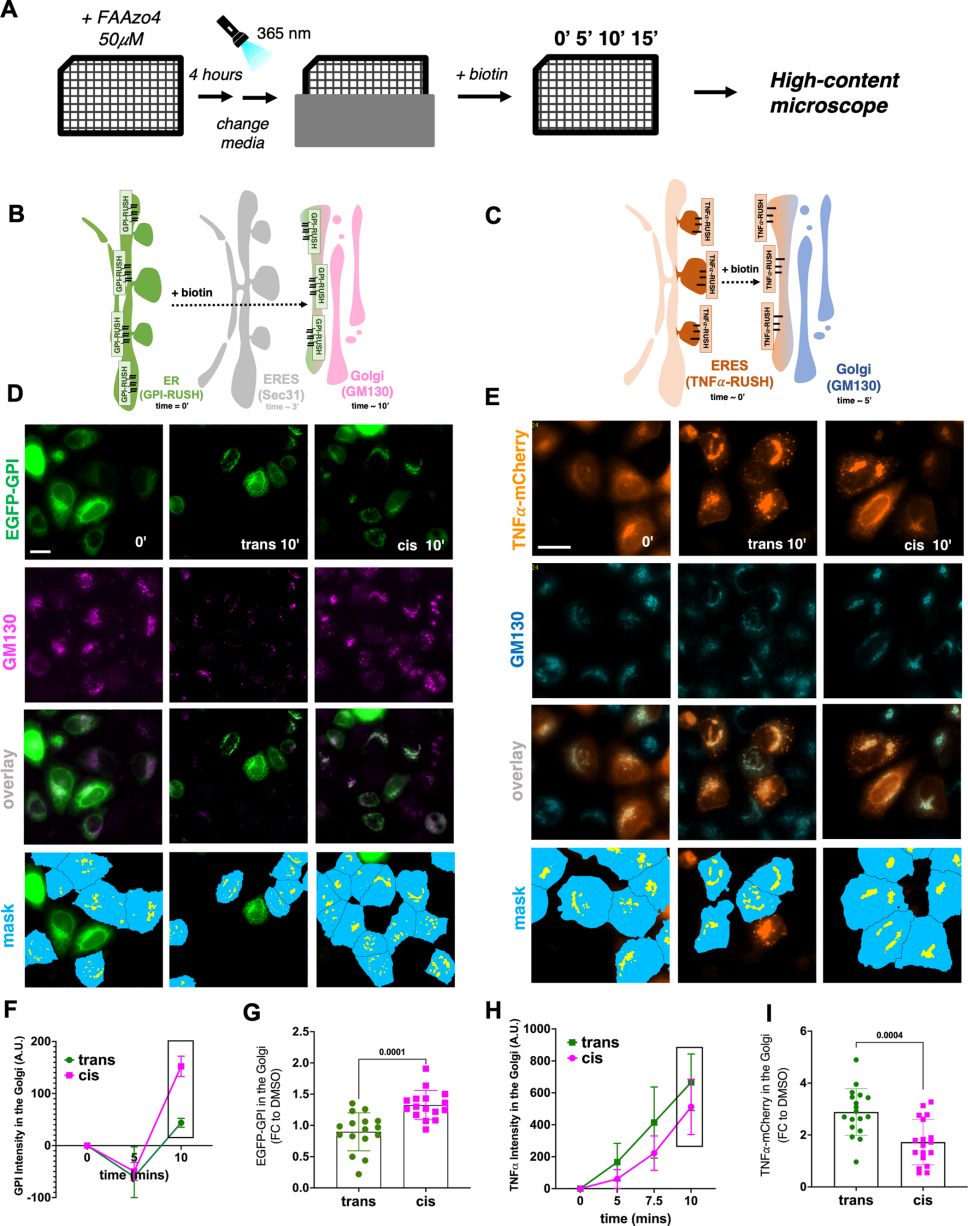
**Establishment of PhotoCells to investigate the role of membrane viscosity in ER-to-Golgi transport**. (A) Scheme describing the experimental set up. **FAAzo4** treatment and “Retention using selective hooks” assay was performed in 96-well plates to allow High Content microscopy. Afterward, **FAAzo4** treatment cells were illuminated with UV-A light at 365 nm and control wells were covered with aluminum foil. Biotin was added at different time points to release the transfected cargos, and after the experiment cells were fixed and prepared for staining and imaging. (B) Schematic representation of the RUSH assay to study ER-to-Golgi transport of a model EGFP-GPI construct. (C) Schematic representation of the RUSH assay to study ER-to-Golgi transport of a TNFa-mCherry construct. (D) Representative microscopy images of the RUSH experiment following GPI-RUSH in HeLa cells. Time points represent the time after biotin addition. Scale Bar: 10 μm. (E) Representative microscopy images of the RUSH experiment following TNF-RUSH in HeLa cells. Scale Bar: 10 μm. (F) Representative curves showing secretion dynamics of GPI-RUSH construct in cells treated with **FAAzo4** before and after irradiation. (G) Effect of light-induced decrease in membrane viscosity on the secretion of the GPI-RUSH construct in HeLa cells. Time = 10 min after biotin addition. Data were normalized to DMSO controls and represented as fold change to the correspondent values. (H) Representative curves showing secretion dynamics TNFα-RUSH construct in cells treated with **FAAzo4** before and after irradiation. (I) Effect of light-induced decrease in membrane viscosity on the secretion of the TNFα-RUSH construct in HeLa cells. Time = 10 min after biotin addition. Data were normalized to DMSO controls and represented as fold change to the correspondent values. *N* = 3; Within each “*n*”, several wells were used for statistical purposes; each point represents the average intensity of a well from the 96 well plate. Statistical analysis was performed by unpaired two-tailed Student’s *t* test with Welch’s correction.

High-content automated microscopy was used to quantitatively analyze the trafficking of proteins from the ER to the *cis*-Golgi, which was labeled by an anti-GM130 antibody (Figure 4A). After incorporation of **FAAzo4** and irradiation to decrease membrane viscosity, we added biotin to release the RUSH constructs and followed the synchronized transport from ER to Golgi (Figure 4B–E). The transport of GPI-RUSH was accelerated, as measured by an increase in the average intensity of EGFP-GPI found in the Golgi area 10 min after biotin addition both in HeLa cells (Figure 4F, G) and HCT116 cells (Figure S5C, D). The opposite effect was observed for TNFα-RUSH, where export was slower after irradiation, resulting in decreased membrane viscosity (Figure 4H, I). These differences might be based on differential localization of these two constructs, as previously mentioned.

To gain deeper insight into this observation and to investigate how decreased membrane viscosity influences the accumulation of proteins at ERES, we performed a RUSH assay measuring the arrival of the GPI-RUSH proteins to ERES, labeled with an anti-Sec31A antibody (Figure 5A–C). The accumulation of EGFP-GPI at SEC31A-labeled ERES was imaged at high resolution using a Leica Stellaris 8 confocal microscope in adaptive lightning mode (Figure 5B, C). Using this protocol, we could see that photoconversion of **FAAzo4** containing lipids and a corresponding decrease in ER membrane viscosity increased concentration of the GPI construct at ERES at time = 3 min while there is no effect on DMSO treated cells (Figures 5C–E and S6B, C).

**Figure 5:**
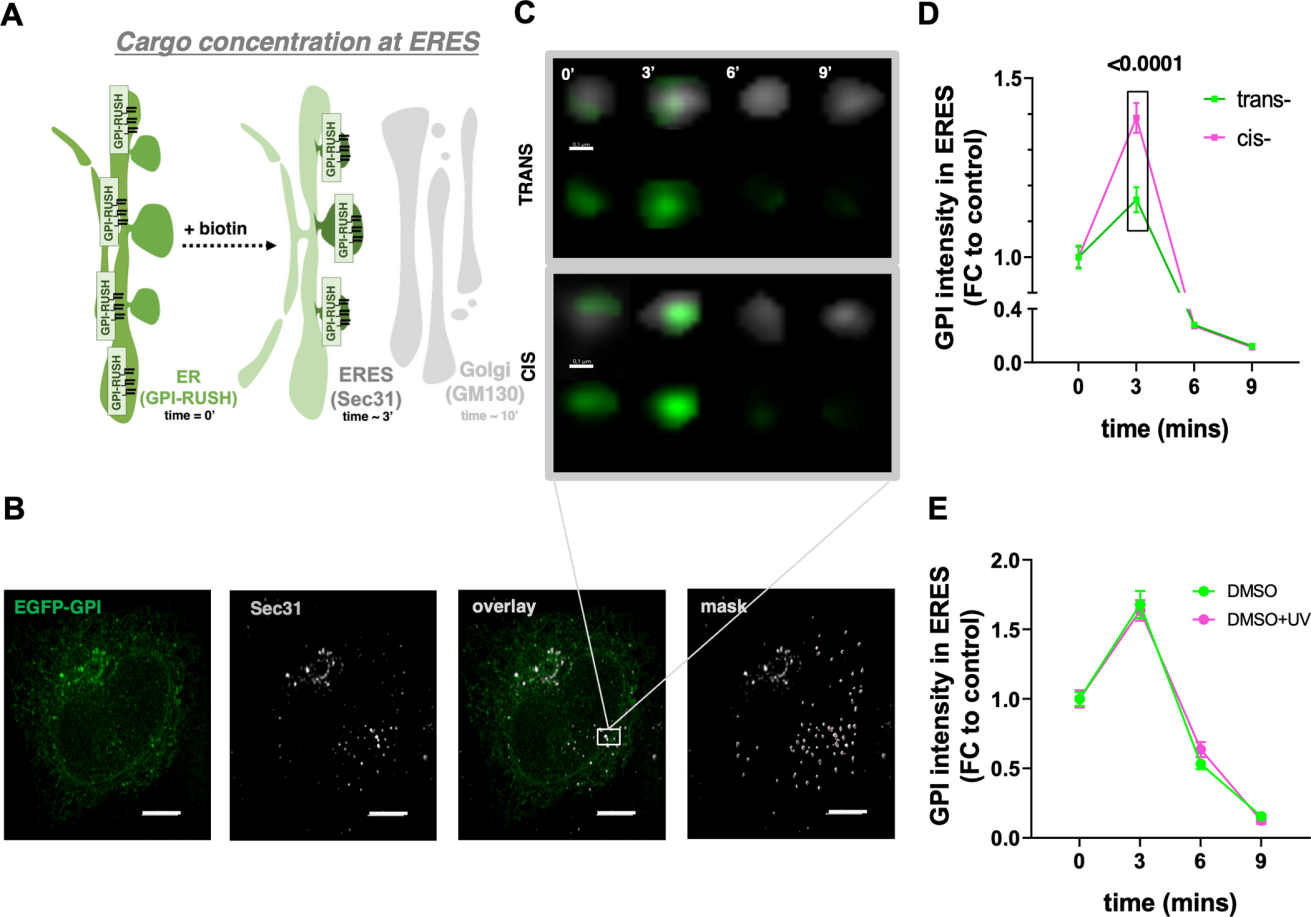
**Membrane viscosity dictates protein recruitment at ERES**. (A) Schematic representation of the RUSH assay to study ER-to-ERES transport of a model EGFP-GPI construct. (B) After high-resolution 3D microscopy, Imaris 9.6.1 software (Bitplane, Zurich, Switzerland) was used to quantify the mean fluorescence intensity of EGFP-GPI within the SEC31A-labeled ERES in HeLa PhotoCells before and after irradiation. Scale Bar: 0.1 μm. (C) Representative image of a HeLa PhotoCell showing GPI-RUSH construct and ERES labeled with an anti-sec31 antibody. Scale Bars: 10 μm. (D) Quantification of GPI-RUSH fluorescent construct located at ERES at different time points after biotin addition in HeLa cells treated with **FAAz4** before and after irradiation. (E) Quantification of GPI-RUSH fluorescent construct located at ERES at different time points after biotin addition in HeLa cells treated with DMSO before and after irradiation.

To study the effect of a decrease in membrane viscosity on the exit from ERES and transport to Golgi we used the same RUSH assay but in combination with a Proximity Ligation Assay (PLA) labeled Sec31 and TNFα-RUSH (Figure 6A). This assay showed that, before biotin addition, TNFα-RUSH is in close proximity to the ERES marker Sec31 thus demonstrating that most of expressed TNFα-RUSH is indeed already prelocalized at ERES (Figures 6B, S5B, lower panel). Figure 6B shows representative images of the PLA assay where increased signal intensities were observed after photoswitching, indicating that TNFα-RUSH stays longer at ERES when the membrane viscosity is decreased. The quantification is shown in Figure 6C for time points of 2.5, 5, 7.5, and 10 min after the release of the construct upon biotin addition. Due to the heterogeneity of cargo expression in the cell population, we chose to measure the accumulation of cargo at ERES after 5 min of biotin addition and the arrival to the Golgi after 10 min of biotin addition as these time points grouped the maximum number of cells with similar trafficking dynamics and data were more reliable (Figure 4H, I). As represented in Figure 6D, the average intensity of TNFα-RUSH at ERES in our Photocells is higher after 5 min of biotin release showing that the export of TNFα-RUSH is slower after irradiation and the subsequent decrease of membrane viscosity.

**Figure 6:**
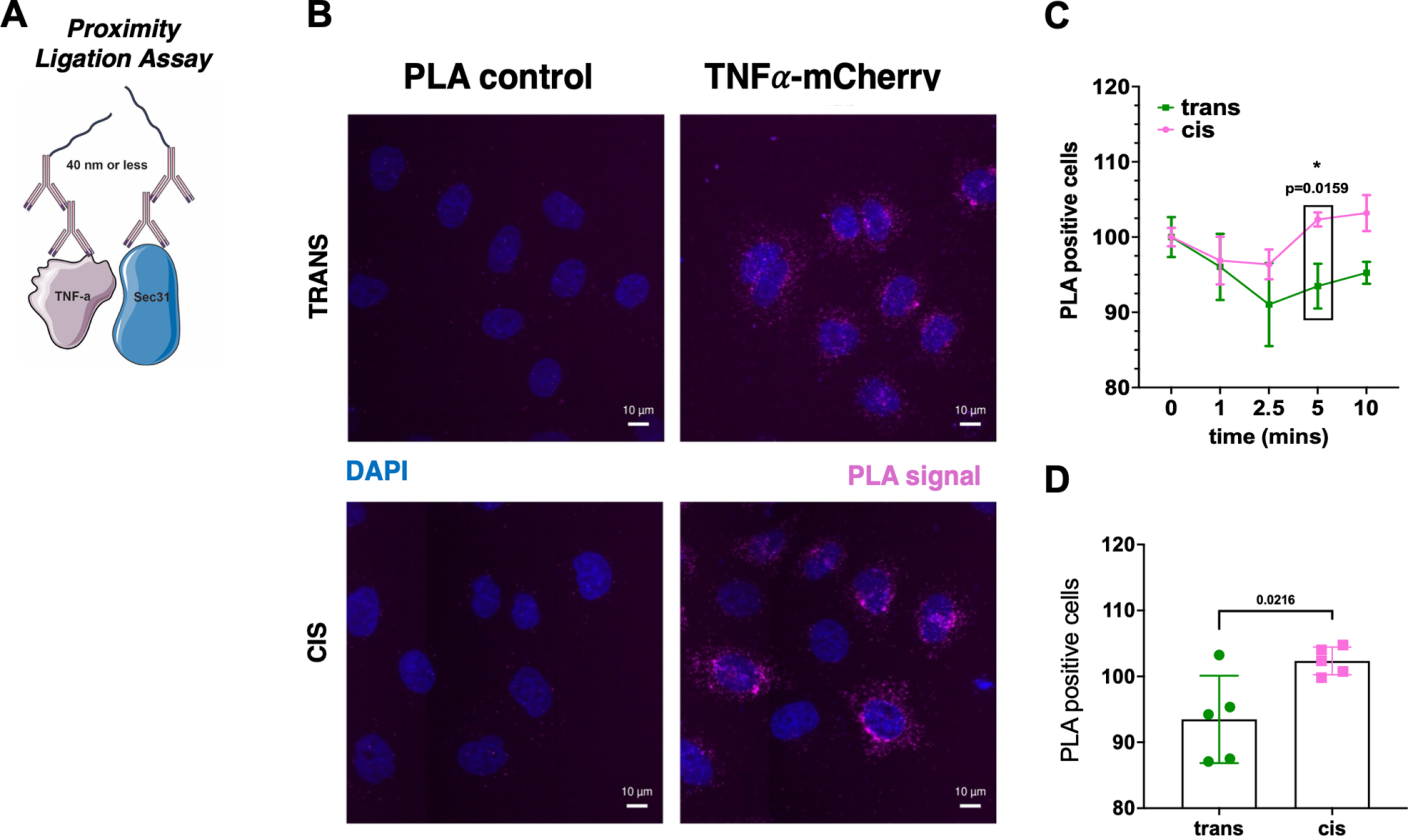
**Decreased membrane viscosity slows down TNFα-RUSH exit from ERES**. (A) Schematic model of the used PLA approach. Images taken from BioRender. (B) Representative images of the PLA approach in HeLa PhotoCells before (trans) and after irradiation (cis). Scale Bars: 10 μm. (C) Quantification of PLA positive PhotoCells before and after irradiation and after biotin addition at different time points. (D) Quantification of PLA positive PhotoCells before and after irradiation after 5 min of biotin addition.

Taken together, our data support differential requirements for membrane viscosity on the ER export process. First, a decrease in membrane viscosity increases the amount of GPI-RUSH protein recruitment into the ERES. Conversely, a decrease in membrane viscosity reduces the rate of export of TNFα from ERES and slows transport to the Golgi.

## Conclusions

In recent years, photoswitchable lipids have been used increasingly for the control of lipid signaling pathways and membrane properties.^12^ While the former was often done in live cells, the modulation of membrane mechanics with photolipids was exclusively studied in reconstituted systems (e.g., GUVs and SLBs). We now show that we can engineer the molecular composition of cellular membranes by integrating photolipids into live cells in a surprisingly simple fashion. This offers unprecedented opportunities to study the contribution of membrane properties, such as viscosity, to cell physiology. Photoswitchable lipids have the advantage that they preserve the integrity of the headgroups of endogenous lipids, allowing them to function similarly to their native analogs, and at the same time, they can rapidly change the physicochemical properties of their lipophilic part.

The engineering of **PhotoCells** was achieved through the feeding of a simple photoswitchable fatty acid analog, termed **FAAzo4**. This precursor is effectively metabolized into phospholipids, mostly **AzoPC** and **AzoPE**, and phospholipid metabolites can be visualized at the endoplasmic reticulum using a clickable **FAAzo4** analog. While uptake and metabolic conversion of **FAAzo4** occurs on the time scale of hours, the photoisomerization induced changes in membrane viscosity occur on the second time scale. As such, this approach allows to distinguish between systemic effects and a biophysical modulation, which could not be addressed with previous approaches, including PUFA-feeding or bulk hydrogenation of unsaturated lipids using palladium catalysis.^38^

We demonstrated that upon illumination and photoconversion of *trans*-**AzoPC** to *cis*-**AzoPC**, the ER membrane viscosity decreases, showing that our approach can be readily combined with other recently developed cell biology techniques to study membrane trafficking. Membrane viscosity and curvature have been proposed to affect several ER-localized processes, such as protein translocation and lipid droplet assembly.^39,40^ Moreover, protein secretion by COPII is known to be modulated by lipid–protein interactions and very likely by changes in the membrane curvature, tension, and asymmetry. In this context, lysolipids have been proposed to facilitate COPII vesicle formation by decreasing the energy required for membrane bending in yeast.^31^ Sphingolipids and ether lipids have been shown to play a role in the secretion of GPI-anchored proteins through specific lipid–protein interactions and likely through affecting membrane biophysical properties.^32,34^ Additionally, the protein export mechanism differs, depending on the selected cargo. It is known that the export of bulky cargos, such as GPI anchored proteins, requires specific COPII protein isoforms and it is also regulated by the cargo crowding itself.^41–43^ Cargos like pro-collagen need distinct adaptors that support their transport into very large noncanonical carriers (TANGO1, cTAGE5) and modulate membrane tension to facilitate elongation of the bud and of transport by acquiring a ring-like structure.^35,44^ Furthermore, a two-step secretion model has recently been proposed by Weigel et al., where they show that the accumulation of the cargo at ERES and export to the Golgi are two independent processes. Moreover, they were able to measure the kinetics of both steps using different cargos.^37^ First, they showed, using the RUSH technology, that TNFα-RUSH is already located at ER exit sites before the release of the cargo and that the first minutes after biotin addition report on the export of the protein to the Golgi. By contrast, in the case of GPI-RUSH, the first minutes after release report on the accumulation of the cargo at ERES. The approach we describe here allowed us to rapidly modify membrane viscosity in the ER and assess the respective membrane viscosity requirements. Decreased membrane viscosity increases the amount of cargo recruited at ERES at early time points, presumably by facilitating protein diffusion. In addition, we showed that decreased membrane viscosity decreased the rate of TNFα ER export from ERES. The reason viscosity affects this step is less clear. Consequently, for proteins that require active concentration at ERES prior to exiting the ER, a decrease in membrane viscosity is advantageous, as it facilitates the initial, and likely rate-limiting, step of the process. Conversely, for proteins that bypass this initial step, decreasing the membrane viscosity impairs the subsequent stage of the protein export process. Thus, the membrane viscosity has a more complex role in the protein export process than previously anticipated. The differential effects on the GPI-anchored protein and TNFα could also reflect heterogeneity in ER exit pathways. In yeast, it has been shown clearly that GPI-anchored proteins are transported through a specialized COPII pathway and that the lipid requirements are different.^37,45^ Due to the high temporal resolution of our approach, we were thus able to establish membrane viscosity as a direct contributor to protein export. Our data support the two-step secretion model and provide insights into the role of membrane viscosity at each step. Importantly, our work demonstrates that a fine-tuning of membrane biophysical properties is necessary to control protein secretion from the ER opening new avenues to investigate and use photopharmacological approaches to modulate the export of disease-related proteins.

The ER is the home of many other cellular processes, including protein folding, protein quality control, the unfolded protein response (UPR), ER associated degradation (ERAD), lipid biosynthesis, and lipid droplet formation, and the ER forms contact sites with all of the other organelles in the cell. PhotoCells should be applicable to the study of membrane viscosity in these processes and, potentially, in other organelles (see the limitation section). They represent a novel approach to gaining direct insight into the role of membranes in biological processes using light as a noninvasive input signal that affords high spatiotemporal control. As such, our approach complements optogenetic methods, which are based on the expression of photoreceptor proteins, and have already been widely employed in cell biology.^46,47^

## Experimental Section

### General Methods

All reagents and solvents were purchased from commercial sources (Sigma-Aldrich, TCI Europe N.V., Strem Chemicals, etc.) and were used without further purification unless otherwise noted. Reactions were monitored by TLC on precoated, Merck Silica gel 60 F_254_ glass backed plates. Flash silica gel chromatography was performed using silica gel (SiO_2_, particle size of 40–63 μm) purchased from Merck. All NMR spectra were measured on a BRUKER Avance III HD 400 (equipped with a CryoProbe). Multiplicities in the following experimental procedures are abbreviated as follows: s = singlet, d = doublet, t = triplet, q = quartet, quint = quintet, sext = sextet, hept = heptet, br = broad, m = multiplet. Proton chemical shifts are expressed in parts per million (ppm, δ scale) and are referenced to the residual protium in the NMR solvent (CDCl_3_: δ = 7.26 CD_3_OD: δ = 3.31). Carbon chemical shifts are expressed in parts per million (δ scale) and are referenced to the carbon resonance of the NMR solvent (CDCl_3_: δ = 77.16; CD_3_OD: δ = 49.00). High-resolution mass spectra (HRMS) were obtained with an Agilent 6224 Accurate Mass time-of-flight (TOF) LC/MS system using either an electrospray ionization (ESI) or atmospheric pressure chemical ionization (APCI) ion source. All reported data refers to positive ionization mode.

### General Lipid Feeding Protocol

Cells were maintained in T25 flasks with medium containing 10% fetal bovine serum and 1% penicillin/streptomycin at 37 °C in a humidified 5% CO_2_ atmosphere. After the cells were seeded and incubated overnight, the medium was carefully removed with a pipet without disturbing the cells. Cells were incubated with **FAAzo4** (10–50 μM in growth medium, prepared from 50 mM DMSO stock) at 37 °C and 5% CO_2_ for 4 h or at indicated time.

### Cell Viability Experiment

To assess cell viability, HeLa and HCT 116 cells were seeded in a 96 well plate (approximately 10k per well in 100 μL) in DMEM/FCS/PS (90:10:1). After 24 h cells were fed with **FAAzo4** according to the standard cell feeding protocol for 4 h and DMSO was used as a control. After 24 h of compound incubation PrestoBlue (Thermo Scientific) was added (20 μL per well), and after 2h fluorescent was measured using a BMG Labtech FLUOstar Omega microplate reader with 544/590 nm filters.

### DNA Transfection

#### HeLa Cells in 96-Well Plate

*Trans*IT-X2 Dynamic Delivery System (Mirus Bio) was used to transfect plasmid DNA (0.05 μg). Briefly, the medium from each well was replaced with 70 μL of fresh media and a 30 μL DNA solution previously diluted in Opti-MEM and mixed with *Trans*IT-X2 Dynamic Delivery System reagent in a 3:1 *Trans*IT-X2 (μL): DNA (μg) ratio. RUSH constructs were a gift from Franck Perez: Str-KDEL_SBP-EGFP-GPI and Str-KDEL_TNF-SBP-mCherry (Addgene plasmid #65294 and #65279).

#### HCT 116 Cells in 96-Well Plate

DNA transfection was done using Lipofectamine 3000 (cat. no. L3000008) following the manufacturer’s instructions. Briefly, DNA-lipid complexes were prepared in Opti-MEM. Separately, a tube was prepared by mixing DNA with P3000 reagent and another tube with Lipofectamine 3000. The content of the tubes was mixed, and 30 μL of the mixture was added per well to the 70 μL of fresh media previously added.

### Lipid Extraction

Lipids were extracted following previously described protocols.^48^ Briefly, cells were washed by cold PBS and scraped off in 500 μL of cold PBS on ice. The suspension was transferred to a 2.0 mL Eppendorf Safe-Lock Tube in which it was spin down at 2,500 rpm for 5 min at 4 °C. After carefully taking off the PBS, samples were extracted following the MTBE protocol.^49^ Briefly, cells were resuspended in 100 μL of water and 360 μL of MeOH and a mixture of internal standards (1 nmol of C17AzoPC, 0.4 nmol of DLPC, 1 nmol of PE31:1, 1 nmol of PI31:1, 3.3 nmol of PS31:1, 2.5 nmol of C12 sphingomyelin, 0.5 nmol of C17 ceramide, and 0.1 nmol of C8 glucosylceramide) was added. Samples were vortexed, following the addition of 1.2 mL of MTBE. The samples were vigorously vortexed at maximum speed for 10 min at 4 °C and incubated for 1 h at room temperature on a shaker. Phase separation was induced by addition of 200 μL of MS-grade water and incubation for 10 min. Samples were centrifuged at 1,000 g for 10 min. The upper phase was transferred into a 13 mm glass tube and the lower phase was re-extracted with 400 μL of a MTBE/MeOH/H_2_O mixture (10:3:1.5, v/v). The extraction was repeated one more time. The combined organic phase was dried with nitrogen flow.

For analysis of the full lipidome, the MTBE extract was divided and one aliquot was deacylated to eliminate phospholipids by methylamine treatment (Clarke method). 0.5 mL monomethylamine reagent (MeOH/H_2_O/*n*-butanol/Methylamine solution (4:3:1:5 v/v) was added to the dried lipid, followed by sonication (5 min). Samples were then mixed and incubated for one h at 53 °C and dried (as above). The monomethylamine treated lipids were desalted by *n*-butanol extraction. 300 μL H_2_O saturated *n*-butanol was added to the dried lipids. The sample was vortexed and sonicated for 5 min, and 150 μL of MS grade water was added. The mixture was vortexed thoroughly and centrifuged at 3200*g* for 10 min. The upper phase was transferred to a 2 mL amber vial. The lower phase was extracted twice more with 300 μL H_2_O saturated *n*-butanol and the upper phases were combined and dried. TL and SL aliquots were resuspended in 250 μL of Chloroform/methanol (1:1 v/v) (LC-MS/HPLC GRADE) and sonicated for 5 min. The samples were pipetted in a 96-well plate (final volume = 100 μL). The TL were diluted 1:4 in negative mode solvent (Chloroform/Methanol (1:2) + 5 mM Ammonium acetate) and 1:10 in positive mode solvent (Chloroform/Methanol/Water (2:7:1 v/v) + 5 mM Ammonium Acetate). The SL were diluted 1:10 in positive mode solvent and infused onto the mass spectrometer. Tandem mass spectrometry for the identification and quantification of lipid molecular species was performed using Multiple Reaction Monitoring (MRM) with a TSQ Vantage Triple Stage Quadrupole Mass Spectrometer (Thermo Fisher Scientific) equipped with a robotic nanoflow ion source, Nanomate HD (Advion Biosciences, Ithaca, NY). The collision energy was optimized for each lipid class. The detection conditions for each lipid class are listed in Table 1. Ceramide species were also quantified with a loss of water in the first quadrupole. Each biological replicate was read in 2 technical replicates (TR). Each TR comprised 3 measurements for each transition. Lipid concentrations were calculated relative to the relevant internal standards and then normalized to the total lipid content of each lipid extract (mol %).

**Table 1:** Detection of Lipids by MS/MS (Related to Lipidome Analyses)

Lipid Class	Standard	Polarity	Mode	*m*/*z* ion	Collision Energy
Phosphatidylcholine [M + H]^+^	DLPC	+	Product ion	184.07	30
Phosphatidylethanolamine [M + H]^+^	PE31:1	+	Neutral ion loss	141.02	20
Phosphatidylinositol [M–H]^−^	PI31:1	–	Product ion	241.01	44
Phosphatidyserine [M–H]^−^	PS31:1	–	Neutral ion loss	87.03	23
Cardiolipin [M–2H]^2–^	CL56:0	–	Product ion	acyl chain	32
Ceramide	C17Cer	+	Product ion	264.34	25
Dihydroceramide	C17DHCer	+	Product ion	266.40	25
Hexacylceramide	C8GC	+	Product ion	264.34	30
Hexacyldihydroceramide	C8GC	+	Product ion	266.40	30
Sphingomyelin	C12SM	+	Product ion	184.07	26

Data analysis was performed using the LipidSig webtool^50^ using Benjamini and Hochberg multiple testing correction, adjusted *p*-value (0.05) and fold change filtering of 1.5.

### UHPLC-HRMS Analyses

Dried samples were resuspended by sonicating in 100 μL of LC-MS-grade chloroform:methanol (1:1, v/v). Reversed-phase UHPLC-HRMS analyses were performed using a Q Exactive Plus Hybrid Quadrupole-Orbitrap mass spectrometer coupled to an UltiMate 3000 UHPLC system (Thermo Fisher Scientific) equipped with an Accucore C30 column (150 mm × 2.1 mm, 2.6 μm) and its 20 mm guard column (Thermo Fisher Scientific). Samples were kept at 8 °C in the autosampler, 10 μL were injected and eluted with a gradient starting at 10% B for 1 min, 10–70% B in 4 min, 70–100% B in 10 min, washed in 100% B for 5 min and column equilibration for an additional 3 min. Eluents were made of 5 mM ammonium acetate and 0.1% formic acid in water (*solvent A*) or in isopropanol/acetonitrile (2:1, v/v) (*solvent B*). Flow rate and column oven temperature were respectively at 350 μL/min and 40 °C. The mass spectrometer was operated using a heated electrospray-ionization (HESI) source in positive and negative polarity with the following settings: electrospray voltage: −3.4 kV (−) or 3.9 kV (+); sheath gas: 51; auxiliary gas: 13; sweep gas: 3; vaporizer temperature: 431 °C; ion transfer capillary temperature: 320 °C; S-lens: 50; resolution: 140,000; *m*/*z* range: 200–1000; automatic gain control: 1e6; maximum injection time: 50 ms. For identification of AzoPC and AzoPE, parallel reaction monitoring (PRM) measurement was performed using a predetermined inclusion list of the corresponding lipid species. The following setting was used in HCD fragmentation: automatic gain control: 1e6; maximum injection time: 25 ms; resolution: 70,000; (N)­CE: 30. Xcaliburv 4.2 (Thermo Fisher Scientific) was used for the data acquisition and processing.

### Click Imaging

HeLa cells from an exponentially growing main culture were detached by trypsinization and seeded on a polylysine precoated imaging dish in 200 μL medium at a density of 25k cells per well. After the cells attached overnight in the incubator at 37 °C and 5% CO_2_, the medium was carefully removed with a pipet without disturbing the cells. After washing with PBS, HeLa cells were incubated with 200 μL of **clFAAzo4** (50 μM in 0.1% DMSO and PBS) at 37 °C and 5% CO_2_ for 4 h. Then, the **clFAAzo4** solution was removed and cells were washed with PBS (1×) before fixation. 200 μL of 4% paraformaldehyde (in PBS) fixation solution was added to each well, and cells were incubated for 20 min at room temperature. Then, the fixation solution was removed and cells were washed with PBS (1×). For the following click-reaction, a master mix was prepared, containing PBS, 5 μM suflo-cyanine-5-azide (from a 5 mM stock in DMSO), 1 mM CuSO_4_ (from a freshly made 20 mM stock in ddH_2_O) and 50 mM sodium ascorbate (from a freshly made 500 mM stock in ddH_2_O). 200 μL of the master mix were added to each well and the cells were incubated in the dark at room temperature for 1 h. After the click-reaction, the labeling solution was removed, and cells were washed with PBS (1×). Subsequently, a solution of ER painter in PBS was added, and cells were incubated at room temperature for 1h and washed again with PBS. Cell imaging was carried out on a Leica DMI6000 B inverted confocal microscope with a Leica HC PL APO 63×/1.30 Glyc CORR CS2 immersion objective to acquire the brightfield and fluorescence images (pinhole 20 μm).

### Epifluorescence Microscopy and Fluorescence Recovery after Photobleaching (FRAP)

#### In Vitro

For epifluorescence measurements of SLBs, an inverted microscope (Olympus IX81 or Nicon Eclipse Ti–U) was used, which is equipped with a 100x oil immersion objective (Olympus UPlanSApo (NA = 1.4) or Nikon SFluor (NA = 1.3–1.5), a mercury short arc (HBO) lamp, and three filter sets for UV-A (λ_exc_ = 330–385 nm, λ_em_ ≥ 420 nm, *P* = 0.28 Wcm^–2^), green (λ_exc_ = 510–550 nm, λ_em_ ≥ 590 nm, *P* = 0.35 Wcm^–2^) and blue (λ_exc_ = 470–490 nm, λ_em_ ≥ 520 nm, *P* = 0.21 Wcm^–2^) light illumination. Image sequences were recorded with a CCD camera (iXon, Andor, exposure times: 0.1–0.2 s).

FRAP measurements were performed on SLBs doped with 1 mol % of dye-labeled lipids (TexasRed-DHPE or Atto465-DOPE). Imaging and photoswitching of the C16-AzoPC SLB were simultaneously achieved by using the UV-A (330–385 nm) and green (510–550 nm) or blue (470–490 nm) filter cubes. A small spot on the SLB was photobleached with intense green/blue light (*P*_510–550 nm_ = 0.97 Wcm^–2^ and *P*_470–490 nm_ = 0.44 Wcm^–2^) for 5 s. The fluorescence recovery was recorded by acquiring an image sequence during exposure with UV-A or green/blue light (exposure times: 0.1–0.2 s). The data were analyzed according to Jönsson et al.^51^

#### In Cellulo

HeLa cells (150000/plate) were seeded in 3.5 cm microscopy plates (IBIDI). Twenty-four h later, cells were transfected with the plasmid Str-KDEL_SBP-mCherry-GPI (Plasmid Addgene #65295) as described in a previous section (DNA transfection protocol). Twenty-four hours later, cells were fed with DMSO or 50 μM FAAzo4 in DMSO and incubated for 4 h prior to imaging. Imaging and FRAP experiments were performed using a Zeiss LSM880 Airyscan confocal microscope with a Zeiss 40× Plan Apochromat NA1.2 water immersion objective. Bright field was used to localize cells, and 2 ROIs of 40 × 20 were selected in each cell, one for laser-bleaching and one to control the photobleaching occurring as a results of the imaging. Bleaching was set up for 5 iterations using a 488 nm laser at 100% power. Under these settings, approximately 50–60% of the signal was bleached. For continuous confocal scanning, the frame mode with 8-bit intensity resolution over 512 × 512 pixels and a pixel dwell time of 1.536 μs was used. EGPI-mCherry was excited at 561 nm and fluorescence recorded at 637 nm every 0.94 s (averaging = 2).

Afterward, for photoconversion of FAAzo4 the same field was illuminated for 5s with UV light at 365 nm using a Zeiss HXP120 lamp with an excitation filter Zeiss Filter set 49 (300–400 nm with a maximum at 365 nm). Right after, the bleaching was repeated, and recovery was recorded as previously. Statistical analysis was performed using PRISM 10. FRAP kinetics curves were adjusted using a two-phase association model, and parameters were obtained from each individual ROI. *N* = 4. Each line represents the average of all the ROIs of all of the replicates and SD.

### Preparation of SUVs and SLBs

**(i) SUVs**. 100 μL of lipids dissolved in CHCl_3_ (c = 6.36 mM) were added to a glass vial and dried using pressure air. The dry lipids were rehydrated in 1.5 mL of ddH_2_O and tip-sonicated (Bandelin Sonoplus) at least two times for 30 s at high intensity until a clear solution was obtained. Finally, the vesicle suspension was centrifuged for 10 min at 8000 rpm. **(ii) SLBs** were made by drop casting SUVs labeled with 1 mol % Texas Red-DHPE or Atto465-DOPE on glass slides to induce vesicle fusion and bilayer formation.^52^ The glass substrates were cleaned via sonication in acetone, propanol, and ddH_2_0, each for 5 min, and additional plasma treatment was performed (PDS-32-G, Harrick Plasma) for 1 min. Prior measurement, the samples were rinsed several times to remove excess pSUVs.

### Retention Using Selective Hooks (RUSH) Assay

#### 1 Accumulation of EGFP-GPI at ERES

HeLa cells were seeded on 12-well plates loaded with 15 mm High Precision #1.5H coverslips and coated with PDL (poly d-lysine, 100 μg/mL per well). The next day, the cells were transfected with the RUSH plasmid Str-KDEL_SBP-EGFP-GPI construct. After 18 h of DNA transfection, the cells were treated with FAAzo4 and exposed to UV-A light at 365 nm as described in the previous sections. Biotin (40 μM) was added at various time points to allow ER export of the EGFP-GPI cargo through the ERES.

Afterward, the plate was fixed using 4% PFA solution for 10 min. Fixed cells were rinsed with PBS and permeabilized in PBS-0.1%Triton X-100–0.1%Tween-20 for 5 min. After permeabilization, cells were washed twice with PBS, and coverslips were transferred to a humidified chamber and blocked with blocking buffer (PBS 0.1% Tween20 with 3% BSA) for 15 min, followed by incubation with anti-SEC31A (1:200 dilution in blocking buffer; BD Transduction Laboratories, 612351) for 90 min. After three washes with PBS, samples were incubated with AlexaFluor680 antimouse IgG (1:500 dilution, Invitrogen A32788) and GFP Booster (1:1000, Chromotek gb2AF488) for 1 h in the dark. Labeled cells were then washed with PBS three times, and absolute EtOH was added for exactly 1 min. The coverslips were then air-dried and mounted onto microscope slides (Epredia AB00000112E01MNZ10) using ProLong Glass Antifade Mountant (Invitrogen P36982). After being cured, the samples were imaged with confocal microscopy.

To image the accumulation of EGFP-GPI on SEC31A-labeled ERES at high resolution, we used a Leica Stellaris 8 confocal microscope and a 63*x*/1.4 NA oil objective (Leica HC PL APO 63×/1.40 OIL CS2) in adaptive lightning mode (in LasX 4.7), setting a high-resolution grade by reducing the pinhole size to 0.8 Airy units. Images were acquired using 499 and 681 nm excitation lasers for EGFP and SEC31A, respectively. Confocal Z-stacks were acquired at high voxel density (42 × 42 × 236 nm).

After high-resolution 3D microscopy, Imaris 9.6.1 software (Bitplane, Zurich, Switzerland) was used to quantify the mean fluorescence intensity of EGFP-GPI within the SEC31A-labeled ERES. To identify the ERES, the Imaris surface detection tool was used with the following parameters Manual threshold: 2,000. Diameter of largest sphere: 0.15 μm; Filter Seed Points: “Quality” above automatic threshold. Filter surfaces were above 10 voxels. ERES objects were used as masks to generate a new channel of EGFP-GPI within the ERES. ERES near the Golgi were discarded from the analysis. Mean EGFP-GPI intensities of individual ERES samples were plotted and analyzed in GraphPad Prism. Data were analyzed by two-way ANOVA with the Benjamini-Hochberg FDR method for multiple comparisons.

#### 2 ER-to-Golgi Transport

For the high content microscopy experiments, we used black 96-well imaging plates (cat. no. 89626, Ibidi). Plates that were used to seed HCT 116 cells were covered with Poly-d-lysine to improve attachment with the following protocol:

10000 HeLa cells or 8000 HCT 116 were seeded per well. HeLa MZ cells were transfected with the RUSH plasmids after 24 h and HCT 116 cells after 48 h to optimize attachment and as described in the previous section. After 24 h of DNA transfection, cells were incubated with FAAzo4 as described in previous sections. After 4 h of treatment, the media in each well was exchanged by fresh media, and cells were left in the incubator before the RUSH experiment for at least 15 min. Next, the 96 well plate was covered with aluminum foil in those wells where the effect of *trans*-FAAzo4 was to be evaluated, and the rest of the wells were illuminated with UV-A light (365 nm) to photoswitch *trans*-FAAzo4 to *cis*-FAAzo4. To synchronize and follow the export of GPI-AP or TNFα in our cells, we used the approach established by Boncompain et al.^36^ and previously described in Jiménez-Rojo et al.^32^ First, a solution of biotin was prepared in a medium at a concentration of 120 μM (40 μM final concentration in each well). The cargo (GPI or TNFα) construct containing an SBP tag (streptavidin binding protein) and a fluorescent protein (EGFP or mCherry) is fused to a minimal ER hook containing streptavidin and a C-terminal ER retention signal (KDEL, Lys-Asp- Glu-Leu) giving rise to the Str-KDEL_SBP-EGFP-GPI or Str-KDEL_TNF-SBP-mCherry construct that is used for transfection. To start the release of the transfected fluorescent cargo, 50 μL of the biotin solution were added at different time points depending on the kinetics of each cargo and on the cell line. Once the biotin solution is added to each well, the reporter is released from the ER hook and follows the secretory pathway. Cells were fixed at different time points (0, 5, 10, and 15 min). Arrival to the Golgi is measured as explained below, following colocalization of the EGFP-GPI or TNFα-mCherry construct with GM130, a Golgi resident protein stained first with a primary purified mouse anti-GM130 antibody (cat. no. 610822, BD Biosciences) following staining with a secondary Alexa Fluor 647-AffiniPure Donkey Anti-Mouse IgG (H+L) (cat. no. 715–605–150, Jackson ImmunoResearch) in combination with EGFP-GPI or Alexa Fluor 488 AffiniPure Donkey Anti-Mouse IgG (H+L) (cat. no. 715–545–151, Jackson ImmunoResearch) in combination with TNFα-mCherry. After the final time point, the plate was fixed using 4% PFA solution and washed with an automated plate washer (BioTek EL406). Cells were then stained as follows: step 1: monoclonal antibody against GM130, 1/500, saponin 0.05%, BSA 1%, in PBS, incubation for 1 h, and step 2: Hoechst 33342 Solution (20 mM) (1/5000), Cy5 or Alexa 488-labeled secondary antibody against mouse IgG, 1/500, incubation for 30 min. Image acquisition was performed immediately after staining using a ImageXpress Micro Confocal High-Content Imaging System (Molecular devices) with the 40× objective. 36 images were captured per well. For image analysis, we used the MetaXpress Custom Module editor to segment the image and generate relevant masks. In the first step, individual cells were identified using staining of the nuclei (Hoechst channel). Next, the Golgi was segmented from the images using the signal coming from the anti-GM130 antibody (Cy5 or Alexa 488 channel). Properly transfected cells were then selected using those with a fluorescence intensity of the cargo ranging between two specific values, identical through all conditions but different depending on the cargo. Finally, the masks were applied to the original fluorescent images, and different measurements were obtained per cell (e.g., integrated intensity, average intensity, and object count). The average intensity value of the fluorescent cargo in Golgi is used to represent the data. The data are shown as a “fold change to DMSO control” where the experimental values have been divided by those of the values obtained in cells treated with DMSO. The same imaging and analysis pipelines were applied to all images. Data analysis was with Prism Graph Pad 9.0, and the statistical analysis was performed by unpaired two-tailed Student’s *t* test with Welch’s correction.

#### 3 Exit from ERES: Proximity Ligation Assay (PLA)

15000 HeLa MZ cells were seeded on black 96-well plates coated with PDL (Poly d-lysine, 100 μg/mL per well). The day after, the cells were transfected with the RUSH Str-KDEL_TNF-SBP-mCherry construct. After 24 h of DNA transfection, cells were incubated with FAAzo4 as described in previous sections. After 4 h of treatment, the media in each well was exchanged by fresh media and cells were left in the incubator before the RUSH experiment for at least 15 min. Next, the 96 well plate was covered with aluminum foil in those wells where the effect of *trans*-FAAzo4 was to be evaluated and the rest of the wells were illuminated with UV-A light (365 nm) to photoswitch *trans*-FAAzo4 to *cis*-FAAzo4. To start the release of the transfected fluorescent cargo, 50 μL of the biotin solution were added at different time points. Once the biotin solution is added to each well, the reporter is released from the ER hook and follows the secretory pathway. After the final time point, the plate was fixed using a 4% PFA solution. To check the colocalization of TNFα with Sec31a, we performed proximity ligation assay (PLA) according to the manufacturer’s recommendation (Sigma). Briefly, we employed oligonucleotide-conjugated secondary antibodies directed against two primary antibodies recognizing Sec31A and the mCherry tag in TNFα. Binding of both antibody species in close proximity allows for their hybridization by connector oligonucleotides, forming a circular DNA strand that can be amplified by PCR. Incorporation of green fluorescence-labeled oligonucleotides in the PCR product enables localized detection of protein interaction. Images were obtained on a PerkinElmer Operetta microscope with the 40× objective. 49 images were captured per well.

Quantification of PLA signals was performed using QuPath, open-source software for bioimage analysis. In the first step, individual cells were identified using the staining of the nuclei (Hoechst channel). Next, a custom classifier was created in QuPath by setting a threshold based on the cell mean intensity to distinguish between positive and negative cells. This classifier was trained and validated to ensure the precise differentiation of cells with significant SEC31-TNFα interactions from those without. The same imaging and analysis pipelines were applied to all images. Data analysis was with Prism Graph Pad 9.0

### Analysis of Gene Transcription

Total RNA from cells was extracted and purified using the RNeasy Mini Kit (Qiagen) and reversely transcribed into cDNA with the iScript cDNA Synthesis Kit (Bio-Rad). Quantitative RT-PCR analysis was performed on a CFX Connect Real-Time PCR system using a SsoAdvanced Universal SYBR Green Supermix (Bio-Rad). The primers used were (5′ → 3′):

- GAPDH Forward: GGC CAT CCA CAG TCT TCT G
- GAPDH Reverse: TCA TCA GCA ATG CCT CCT G
- CHOP Forward: AGA ACC AGG AAA CGG AAA CAG A
- CHOP Reverse: TCT CCT TCA TGC GCT GCT TT

Fold change in transcript levels was calculated using the cycle threshold (CT) comparative method (2^–ddCT^) normalizing to CT values of internal control genes *GAPDH*.

### Nanostring Analysis

For the quantification of genes related to cell stress, 100 ng of total RNA was extracted from cells post-treatment with FAAzo4 at different concentrations and conformations by using the RNeasy Mini Kit. The RNA samples were then analyzed using the NanoString nCounter system with a custom panel targeting genes involved in the Unfolded Protein Response (UPR) and Sterol Regulatory Element-Binding Proteins (SREBP) pathways. This system employs unique molecular barcodes and hybridization techniques to directly count individual target RNA with highly accurate and reproducible quantification. The data were normalized and analyzed according to NanoString’s standard protocols using nSolver software.

### Generation of ACSL4 Mutant Cells

ACSL4 polyclonal HeLa MZ mutant cells were generated with CRISPR-Cas9, using the highly efficient cotargeting strategy GENF^53^ (GEne cotargeting with Non-eFficient conditions) as following. A plasmid for mammalian cell expression of ACSL4-targeting single-guide RNA (sgRNA) and Cas9 was constructed by annealing the oligonucleotides caccg­TCATGG­GCTAAA­TGAA­TCTG (target sequence in upper case) and aaac­CAGATT­CATTTA­GCCCA­TGAc and ligating them with Quick Ligase (New England Biolabs) into pX330 plasmid (Addgene no. 42230, deposited by Feng Zhang) cleaved with FastDigest BpiI (Thermo Scientific). This plasmid (495 ng) was cotransfected with 5 ng of a previously generated mismatched sgRNA expression plasmid^53^ to target HPRT1 (target sequence with mismatches in lower cases: gtGC­CCTCTG­TGTGC­TCAA) using Lipofectamine 3000. Five days after transfection, mutant cells were selected with 6 μg/mL 6-thioguanine for 1 week, which kills cells with a functional HPRT1 gene. 6-thioguanine selected cells having mutated HPRT1 despite the use of mismatched sgRNA and the low amount of plasmid used to express it, enabling the enrichment of cells with high CRISPR-Cas9 activity. The targeted ACSL4 region was amplified from control cells and mutated cells with the PCR primers ACTGAT­TGCATG­CTGTG­AATCT and GGTGTG­GAGGTC­ACCAA­TCAC, the amplicons were treated with Exonuclease I and FastAP Thermosensitive Alkaline Phosphatase (both from Thermo Scientific) and used for Sanger sequencing (Fasteris) with the sequencing primer CAGCTA­TTAAAC­TTAAG­CCTGC. Mutation efficiency was assessed by analyzing sequence traces with TIDE^54^ (Tracking Indels by DEcomposition). This led to a polyclonal population having undetectable levels of nonmutated ACSL4 alleles.

### Pe-GFP-VSV Infection Assay

The VSV infection assay was performed using previous published protocols.^55^ We used the same materials and instrument as the RUSH assay unless indicated below. Briefly, 12,000 HeLa cells/well were seeded in 96-well plates using Fluoro-Brite DMEM supplemented with 10% FCS and 2 mM l-Glutamine. After 20 h, cells were incubated with 50 μM FAAzo4 or DMSO for 4 h. Next, the 96 well solid black plate was partially covered with aluminum foil for the wells where the effect of *trans*-FAAzo4 was to be evaluated, and remaining wells were illuminated with UV-A light (365 nm) to photoswitch *trans*-FAAzo4 to *cis*-FAAzo4. After illumination, cells were washed with cold PBS (3x), supplemented with 100 μL of cold VSVmem (Glasgow Minimum Essential Medium, 10 mM TES, 10 mM MOPS, 15 mM HEPES, 2 mM NaH2PO4, 35 mg/L NaHCO3, pH = 7.4), and incubated for 5 min on ice. Cells were then treated with 50 μL of Pe-GFP-VSV virus to reach a final concentration of 0.5 MOI, incubated for 45 min on ice with gentle shaking, washed by PBS (3×), supplemented with 150 μL of growth medium (37 °C), and incubated for another 3.5 h before fixation using 3% paraformaldehyde. For staining, the plate was washed (3×) by PBS, and cells were incubated with Hoechst in PBS for 30 min and washed again (3×). Image acquisition was performed immediately after staining using an ImageXpress Micro Confocal High-Content Imaging System (Molecular devices) with the 20× objective. For image analysis, we used the MetaXpress Custom Module editor software to first segment the image and generate relevant masks. Cells were scored as infected by the presence of the Pe-GFP-VSV protein by automated image analysis.

### Phophoproteomics

#### Cell Lysis and Protein Digestion

Cell pellets were suspended in lysis buffer composed of 8 M urea and 100 mM TRIS (pH, 8.5) and lysed by sonication. Protein concentrations were measured by the BCA protein assay. Lysates were supplemented with TCEP (final 5 mM) and chloroacetamide (final 10 mM) and incubated at 56 °C for 1h. After 6× dilution with 25 mM ammonium bicarbonate, proteins were digested with trypsin (100:1 ratio, o/n @ 37 °C). Digestion was stopped by acidification with FA (final 0.5%), and peptides were desalted on tC18 cartridges (50 mg, 1 cm^3^, Waters) according to manufacturer instructions. Peptide elution was performed in 400 μL of 40% ACN and 200 μL of 60% ACN w/o any acid. Peptide concentrations were measured by Pierce colorimetric peptide assay; subsequently, these measurements were used for TMT labeling. Finally, all samples were dried in speedvac and stored at −80 °C.

#### TMT Labeling

Each peptide sample were reconstituted in 20 μL of 50 mM HEPES, pH = 8.5. TMT labeling was performed with TMTPro isobaric tags according to procedure adapted from.^56^ Briefly, samples were labeled with 8 μL of the corresponding TMT label ACN stock (12.5 μg/μL of label). Samples were incubated at RT for 30 min before quenching with 40 μL of 500 mM ABC (15 min at 37 °C). To lower ACN concentration prior to the desalting step, each sample was diluted with an additional 400 μL of 0.5% TFA. All TMT channels were pooled together and desalted on tC18 cartridge (50 mg, 1 cm^3^, Waters) according to manufacturer instructions. Small aliquots were used as QC for labeling completion. Eluates were dried in a speedvac concentrator and stored at −80 °C.

#### Phosphopeptide Enrichment

Phosphorylated peptides were enriched by IMAC on high-selectivity Fe-NTA spin columns (Thermo Scientific) according to the manufacturer instructions. Eluted pSTY peptides were dried in a speedvac concentrator and resolubilized in 10 μL of 2% ACN and 0.5% AcOH prior to LC-MS/MS analysis.

#### LC-MS/MS

LC separation was performed online on an EASY-nLC 1000 (Thermo Scientific) utilizing an Acclaim PepMap 100 (75 μm × 2 cm) precolumn and a PepMap RSLC C18 (2 μm, 100A × 50 cm) analytical column. Peptides were gradient eluted from the column directly into an Orbitrap HFX mass spectrometer using 136 min ACN gradient from 5 to 26% B in 100 min followed by ramp to 40% B in 20 min and final equilibration in 100% B for 15 min (A = 2% ACN 0.5% AcOH/B = 80% ACN 0.5% AcOH). Flow rate was set at 200 nL/min. High resolution full MS spectra were acquired with a resolution of 120,000, an AGC target of 3 × 10^6^, a maximum ion injection time of 100 ms, and scan range of 400 to 1600 *m*/*z*. Following each full MS scan, 20 data-dependent HCD MS/MS scans were acquired at a resolution of 60,000, AGC target of 5 × 10^5^, maximum ion time of 100 ms, one microscan, 0.4 *m*/*z* isolation window, NCE of 30, fixed first mass 100 *m*/*z* and dynamic exclusion for 45 s. Both MS and MS^2^ spectra were recorded in profile mode.

#### Data Analysis

MS data were analyzed using MaxQuant software version 1.6.3.4 ^57^ and searched against the SwissProt subset of the human uniprot database (http://www.uniprot.org/) containing 20,430 entries. Database search was performed in Andromeda^58^ integrated in MaxQuant environment. A list of 248 common laboratory contaminants included in MaxQuant was also added to the database, as well as reversed versions of all sequences. For searching, the enzyme specificity was set to trypsin, with the maximum number of missed cleavages set to 2. The precursor mass tolerance was set to 20 ppm for the first search used for nonlinear mass recalibration^59^ and then to 6 ppm for the main search. Phosphorylation of S/T/Y and oxidation of methionine were searched as variables; carbamidomethylation of cysteines was searched as a fixed modification. TMT labeling was set to lysine residues and N-terminal amino groups, and corresponding batch-specific isotopic correction factors were accounted for. The false discovery rate (FDR) for peptide, protein, and site identification was set to 1%, and the minimum peptide length was set to 6. Subsequent data analysis were performed in either Perseus^60^ (http://www.perseus-framework.org/) or using R environment for statistical computing and graphics (http://www.r-project.org/).

## Supplementary Material



## Notes

Figures S1–S6 presenting extended data that complement the main text, including additional experimental validation, quantification analyses, and control studies; ^1^H and ^13^C NMR spectra as well as experimental information for newly synthesized compounds (PDF)
