# Supplementary material for: Optical Control of Membrane Viscosity Modulates ER-to-Golgi Trafficking
Source: ACS Cent Sci. 2025 Aug 12;11(9):1736–52. doi: 10.1021/acscentsci.5c00606 (PMC12464753; doi:10.1021/acscentsci.5c00606)
Supplement: Supplementary file 1 [file oc5c00606_si_001.pdf]

Supporting Information - Optical Control of Membrane Viscosity Modulates ER-to-Golgi Trafficking

Noemi Jiménez-Rojo[1,2,3,15], Suihan Feng[1,4,15], Johannes Morstein[5,6,15]*, Stefanie D. Pritzl[7,8], Antonino Asaro[1,9], Sergio López[10], Yun Xu[4], Takeshi Harayama[1,11], Nynke A. Vepřek[5,12], Christopher J. Arp[5], Martin Reynders[5,6], Alexander J. E. Novak[5], Evgeny Kanshin[13], Jan Lipfert[8,14], Beatrix Ueberheide[13], Manuel Muñiz[10], Theobald Lohmüller[7], Howard Riezman[1]*, Dirk Trauner[5]*

[1]NCCR Chemical Biology, Department of Biochemistry, University of Geneva, Geneva, Switzerland
[2]Department of Biochemistry and Molecular Biology, University of the Basque Country (UPV/EHU), 48940 Leioa, Spain.
[3]Ikerbasque, Basque Foundation for Science, 48013 Bilbao, Spain.
[4]Unit of Chemical Biology and Lipid Metabolism, Key Laboratory of Immune Response and Immunotherapy, Shanghai Institute of Immunity and Infection, Chinese Academy of Sciences, Shanghai, China
[5]Department of Chemistry, New York University, New York, New York, United States
[6]Division of Chemistry and Chemical Engineering, California Institute of Technology, Pasadena, California 91125, United States
[7]Chair of Photonics and Optoelectronics, Nano-Institute Munich, Department of Physics, Ludwig Maximilian University, Munich, Germany
[8]Soft Condensed Matter and Biophysics, Department of Physics and Debye Institute for Nanomaterials Science, Utrecht University, Utrecht, the Netherlands
[9]Institute of Bioengineering (IBI) and Global Health Institute, École polytechnique fédérale de Lausanne (EPFL), Lausanne, Switzerland.
[10]Department of Cell Biology, Faculty of Biology, University of Seville and Instituto de Biomedicina de Sevilla (IBiS), Hospital Universitario Virgen del Rocío/CSIC/Universidad de Sevilla, 41012 Seville, Spain.
[11]Institut de Pharmacologie Moléculaire et Cellulaire, CNRS, Université Côte d'Azur - CNRS UMR1715 - Inserm U1323, Valbonne, France.
[12]Department of Chemistry, Ludwig Maximilian University, Munich, Germany
[13]NYU Grossman School of Medicine, New York, New York, USA
[14]Institute for Physics, Augsburg University, Augsburg, Germany
[15]N.J.R, S.F., and J.M. contributed equally to this study and have the right to list themselves first in bibliographic documents.
*Correspondance: morstein@caltech.edu (J.M.), howard.riezman@unige.ch (H.R.), dtrauner@upenn.edu (D.T.)

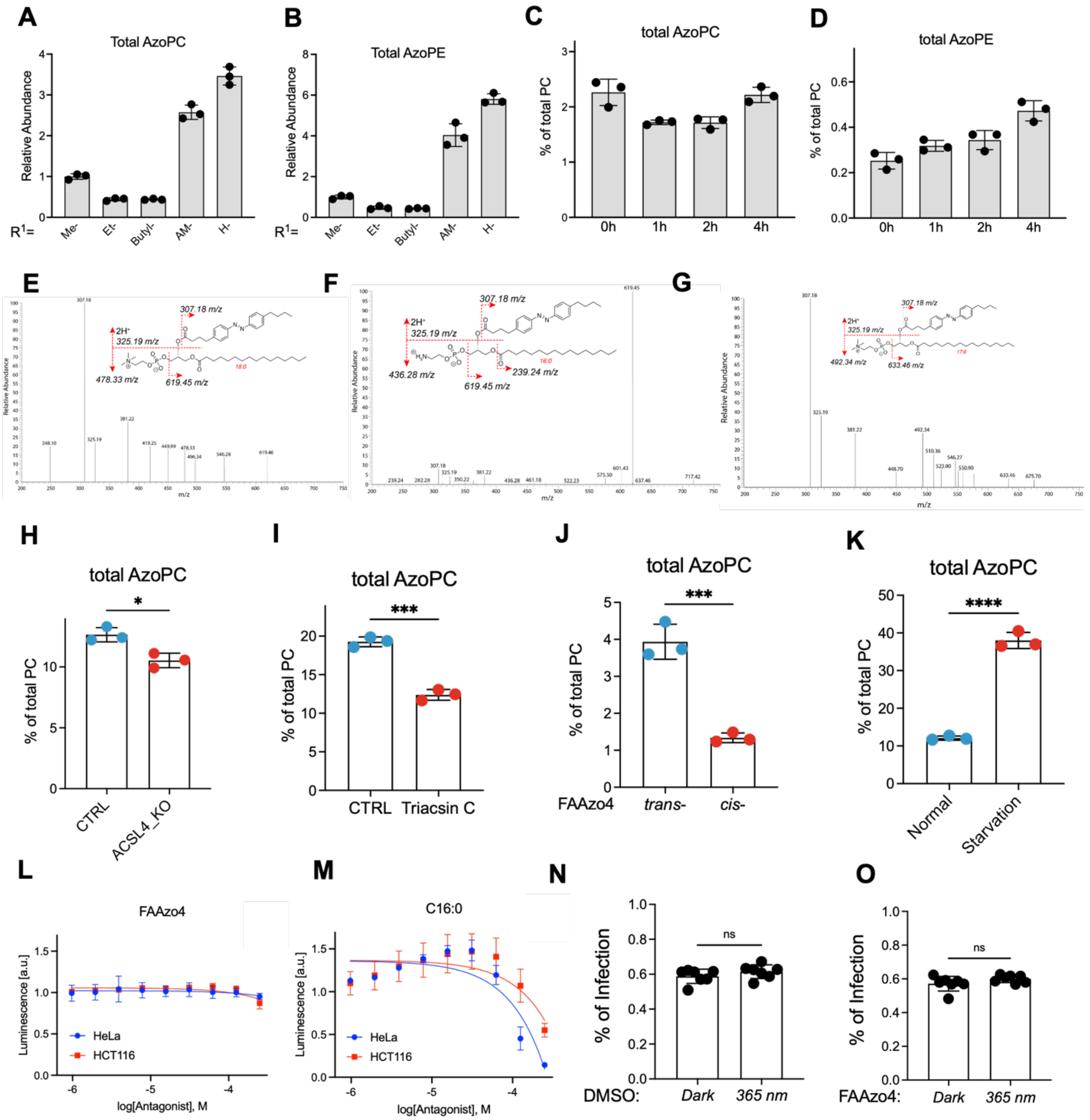

Figure S1 l LCMS characterization of C17-AzoPC and cell viability of HeLa PhotoCells. (A, B) Incorporation of **FAAzo4** into **AzoPC**s and **AzoPE**s after treatment of **FAAzo4** esters or **FAAzo4** in HeLa cells. (C, D) HeLa cells were incubated with **FAAzo4** for 30 minutes, replaced by fresh medium, and then collected at indicated time points. (E,F,G) LC-MS/MS spectra of ions (m/z) correspond to C16:0-**AzoPC** and C16:0-**AzoPE** and **C17-AzoPC** respectively. The putative structures of C16:0-**AzoPC** and C16:0-**AzoPE** are presented above the corresponding spectra. (H) HeLa cells were incubated with **FAAzo4** in control or ACSL4 KO cells. (I) HeLa cells were incubated with **FAAzo4** in the presence/absence of 10 µM Triacsin C. (J) HeLa cells were incubated with *trans-* or *cis-* **FAAzo4**. (K) After 24-hour starvation, HeLa cells were incubated with **FAAzo4** and

compared to cells in normal growth conditions.(L,M) Cell viability of HeLa cells after treatment with **FAAzo4** or palmitic acid in a dose-dependent fashion for 72 h. (N, O) **Light-dependent VSV infection assay in HeLa PhotoCells. FAAzo4** or DMSO treated cells were illuminated with UV-A light at 365 nm and control wells were covered with aluminum foil. After the infection experiments, cells were fixed, stained with Hoechst, and images were acquired using automatic fluorescence microscopy.

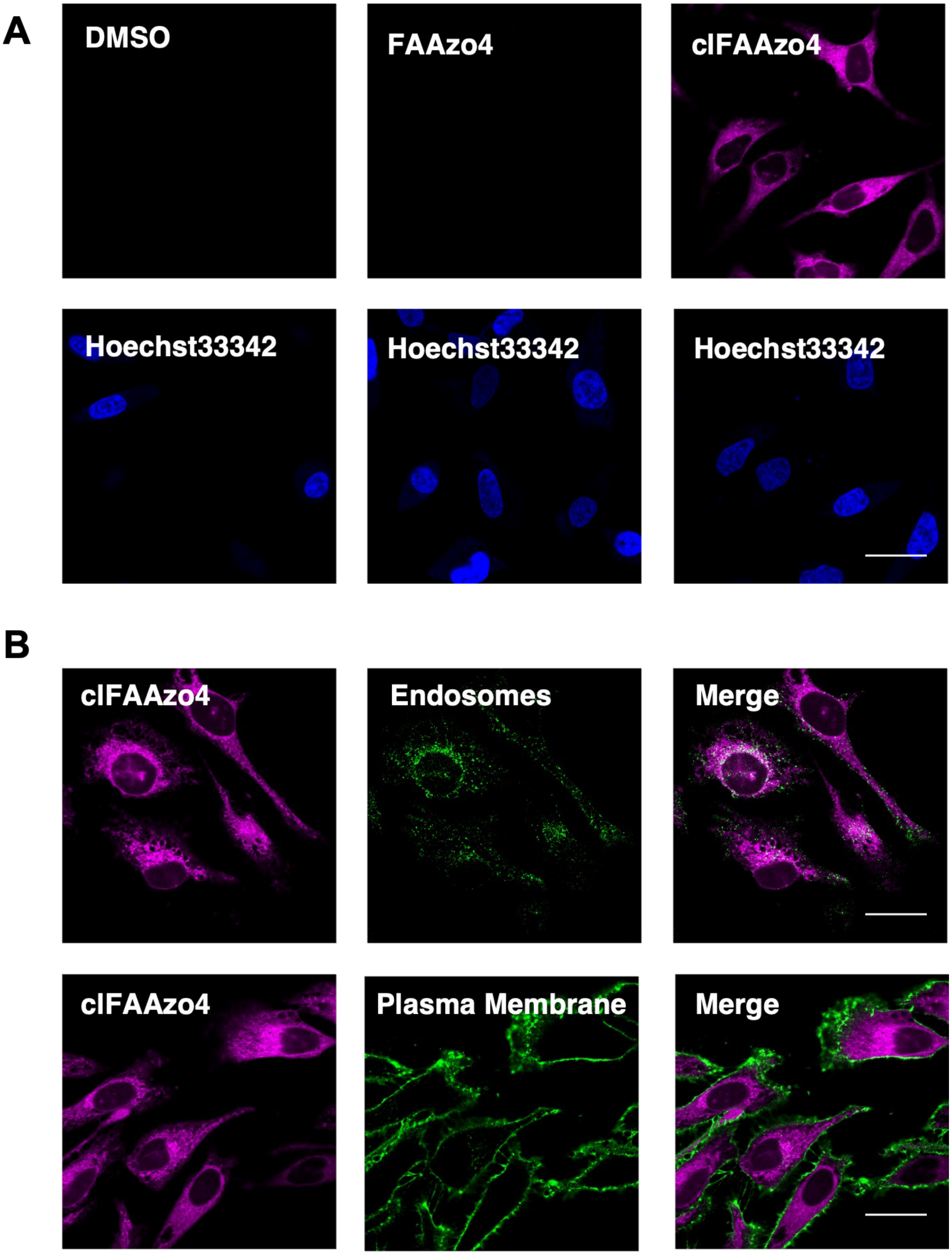

Figure S2 I Click-imaging of alkyne-modified FAAzo4 Colocalization and Control Experiments. (A) Control Experiments for Click-Imaging with DMSO and non-clickable FAAzo4 samples fixed and incubated with sulfo-cyanine-5-azide. Confocal images (63 x) were obtained using λ_{Ex} = 646 nm for Cy5 and λ_{Ex} = 405 nm for Hoechst33342. (B) Co-staining experiments for endosomes (Alexa Fluor™ 488-Transferrin Conjugate) and

plasma membrane (Alexa Fluor™ 488-Wheat Germ Agglutinin Conjugate). Confocal images (63 x) were obtained using λ_{Ex} = 646 nm for Cy5 and λ_{Ex} = 488 nm for AlexaFluor™ 488. Scale Bar: 20 μm.

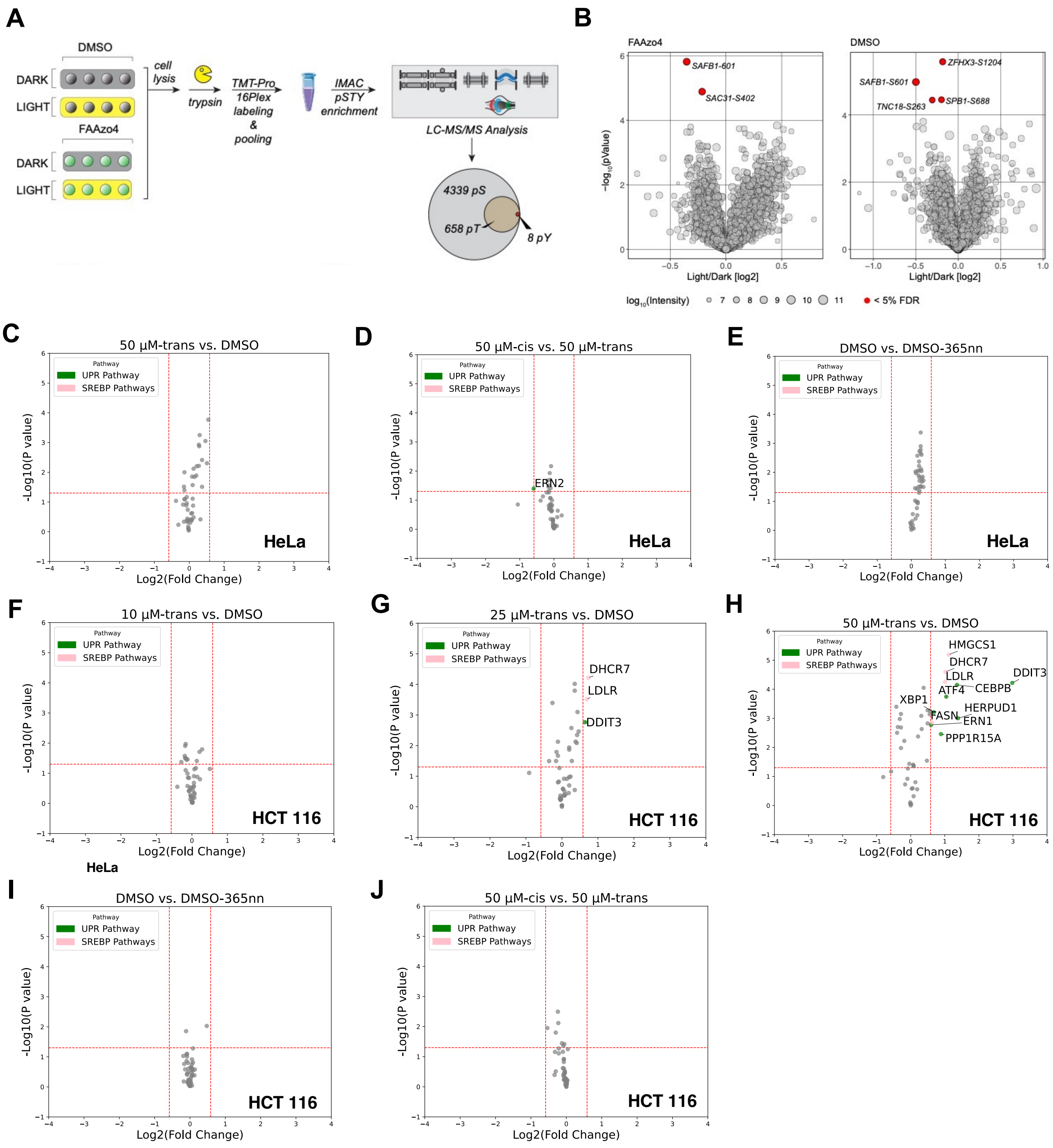

Figure S3 I Characterization of FAAZo4 incorporation in HeLa and HCT 116 PhotoCells. Related to Figure 2. (A) Light-dependent phosphoproteomics in HeLa PhotoCells. **FAAzo4** and control HeLa cell pellets from both dark/light-stimulated conditions (n=4 from each group) were processed in parallel. Cells were lysed in urea buffer and proteins were digested into peptides with trypsin. Peptides from each sample were labeled with TMT-Pro isobaric tags and all samples were pooled together.

Phosphopeptides were purified by IMAC and subsequently analyzed by LC-MS/MS, which resulted in identification and quantification of ~ 5,000 phosphosites. (B) Statistical analyses revealed only a minor difference between light/dark conditions (permutation-based FDR threshold of 5%). We did not observe more difference within **FAAzo4**-trated cells compared to controls. (C-J) Quantification of the expression of genes related to cell stress in HeLa and HCT PhotoCells post-treatment with FAAzo4 at different concentrations and conformations. The RNA samples were then analyzed using the NanoString nCounter® system with a custom panel targeting genes involved in the Unfolded Protein Response (UPR) and Sterol Regulatory Element-Binding Proteins (SREBP) pathways.

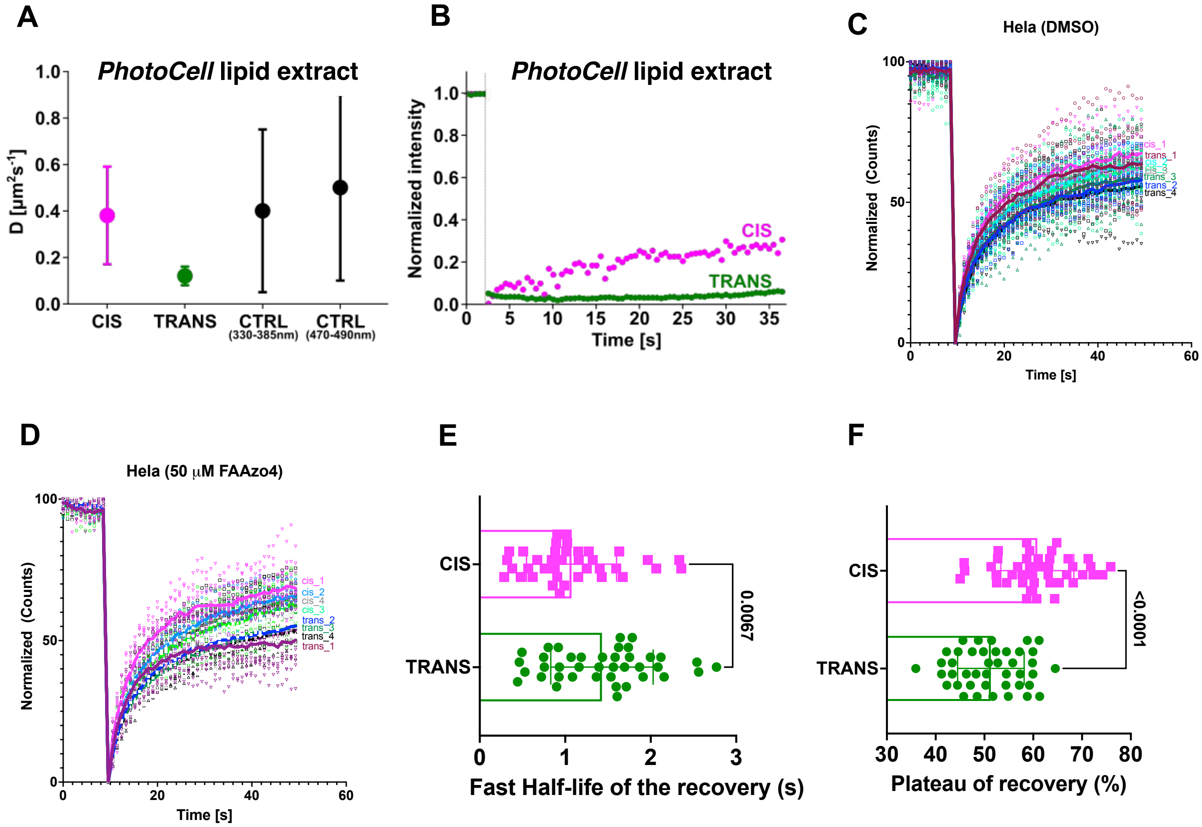

Figure S4 I Biophysical modulation of FAAzo4 containing cellular membranes. Related to Figure 3. (A) Diffusion coefficients of fluorescent lipids in vesicles formed from Photocell lipid extracts + 1 mol% Texas Red-DHPE. (B) Diffusion kinetics of fluorescent lipids in vesicles formed from Photocell lipid extracts + 1 mol% Texas Red-DHPE. (C, D) FRAP kinetics of of individual biological replicates of GPI-mCherry construct in HeLa PhotoCells treated with DMSO and FAAzo4 before and after irradiation. Each line represents the average of ~15 cells/ROIs. N=4. FRAP kinetics curves were adjusted using a two-phase association model and parameters were obtained from each individual ROI. N=4. Each line represents the average of all the ROIs of all the replicates and SD. (E) Half-life of the fluorescence recovery. (F) % of the fluorescence recovered after reaching the plateau.

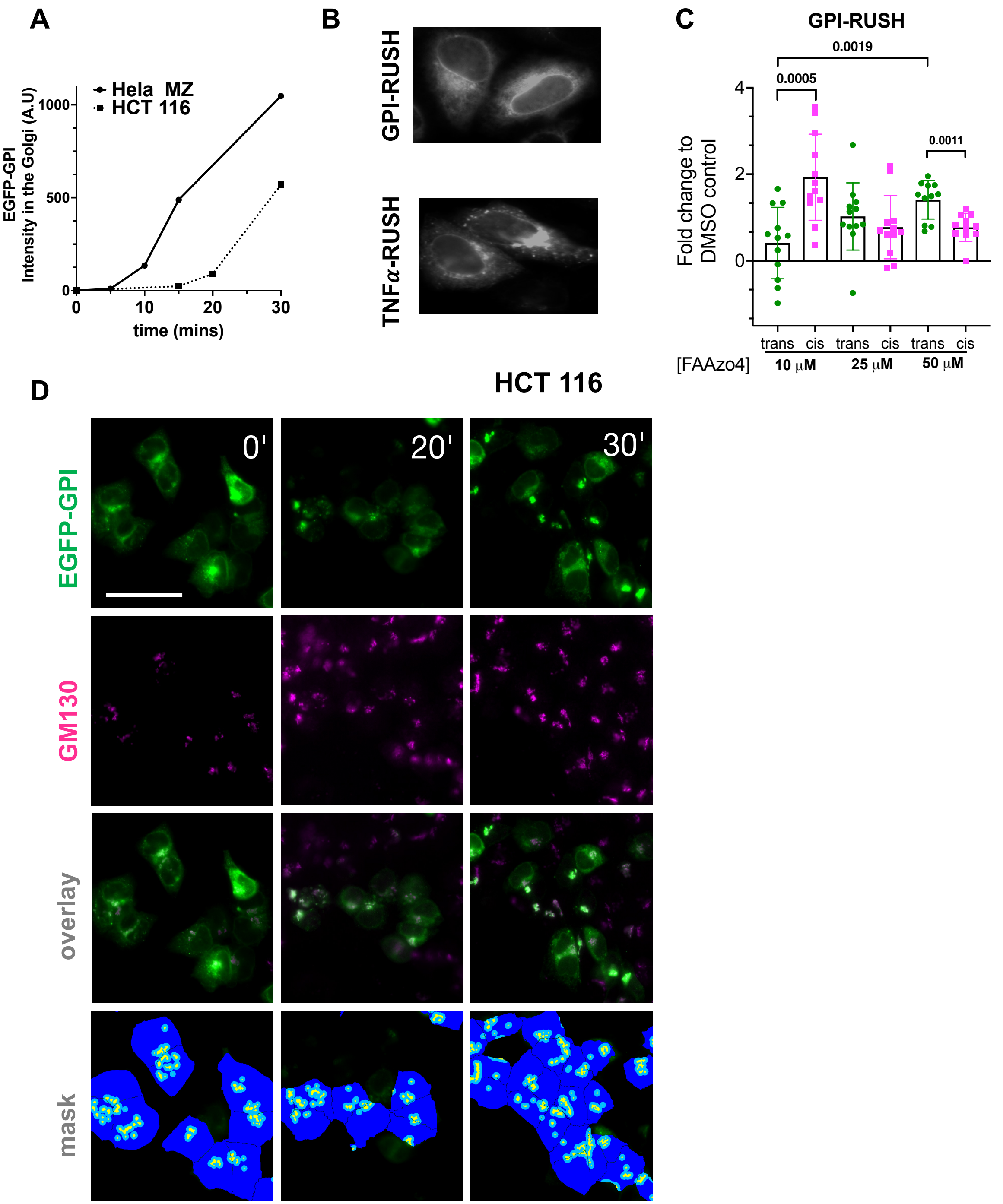

Figure S5 I RUSH assay in HeLa and HCT 116 PhotoCells. Related to Figure 4. (A) Representative curves of the RUSH experiment following GPI-RUSH in HeLa cells and HCT 116 cells. Time points represent the time after biotin addition. (B) Representative images showing expressed selected cargo in HeLa cells before release upon biotin addition. Scale Bar: 10 μm. (C) Effect of light-induced increase in membrane viscosity on the export of the GPI-RUSH construct in HCT 116 cells treated with different amounts of **FAAzo4**. (D) Representative microscopy images of the RUSH experiment following GPI-

RUSH in HeLa cells. Time points represent the time after biotin addition. Scale Bar: 20 μm.

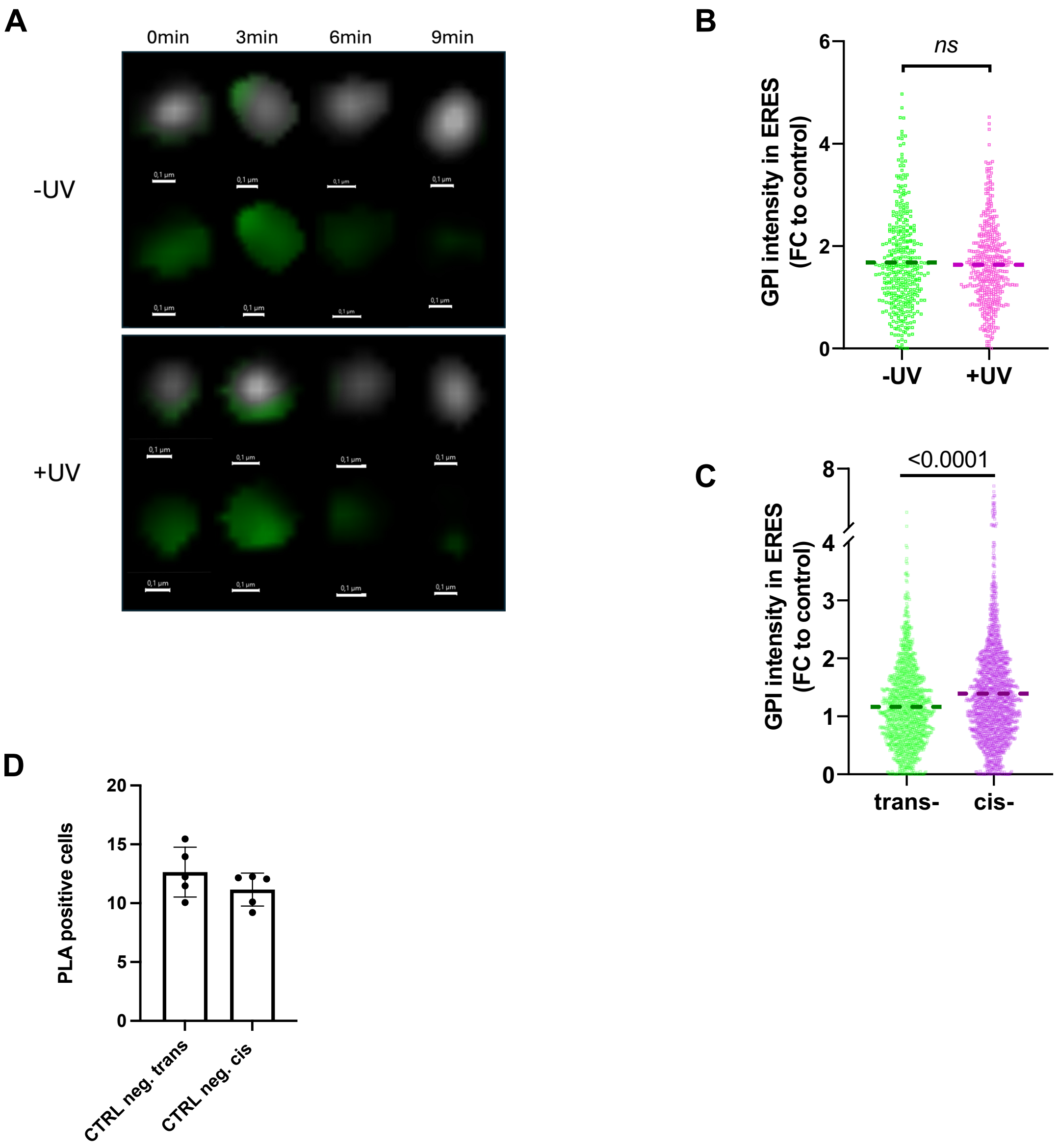

Figure S6 I Quantification of EGFP-GPI construct at ERES. Related to Figure 5. (A) After high-resolution 3D microscopy, Imaris 9.6.1 software (Bitplane, Zurich, Switzerland) was used to quantify the mean fluorescence intensity of EGFP-GPI within the SEC31A-labeled ERES in control HeLa cells treated with vehicle (DMSO) before and after irradiation. Scale Bar: 0,1 µm (B) Quantification of EGFP-GPI intensity at ERES (Sec31) in control HeLa cells treated with DMSO after 3 minutes of biotin addition. Each dot represents an ERES. N>1500. (C) Quantification of EGFP-GPI intensity at ERES (Sec31) in HeLa cells treated with FAAzo4 after 3 minutes of biotin addition. Each dot represents an ERES. N>1500. (D) Quantification of PLA positive HeLa PhotoCells treated with DMSO before and after irradiation.

Synthetic Scheme

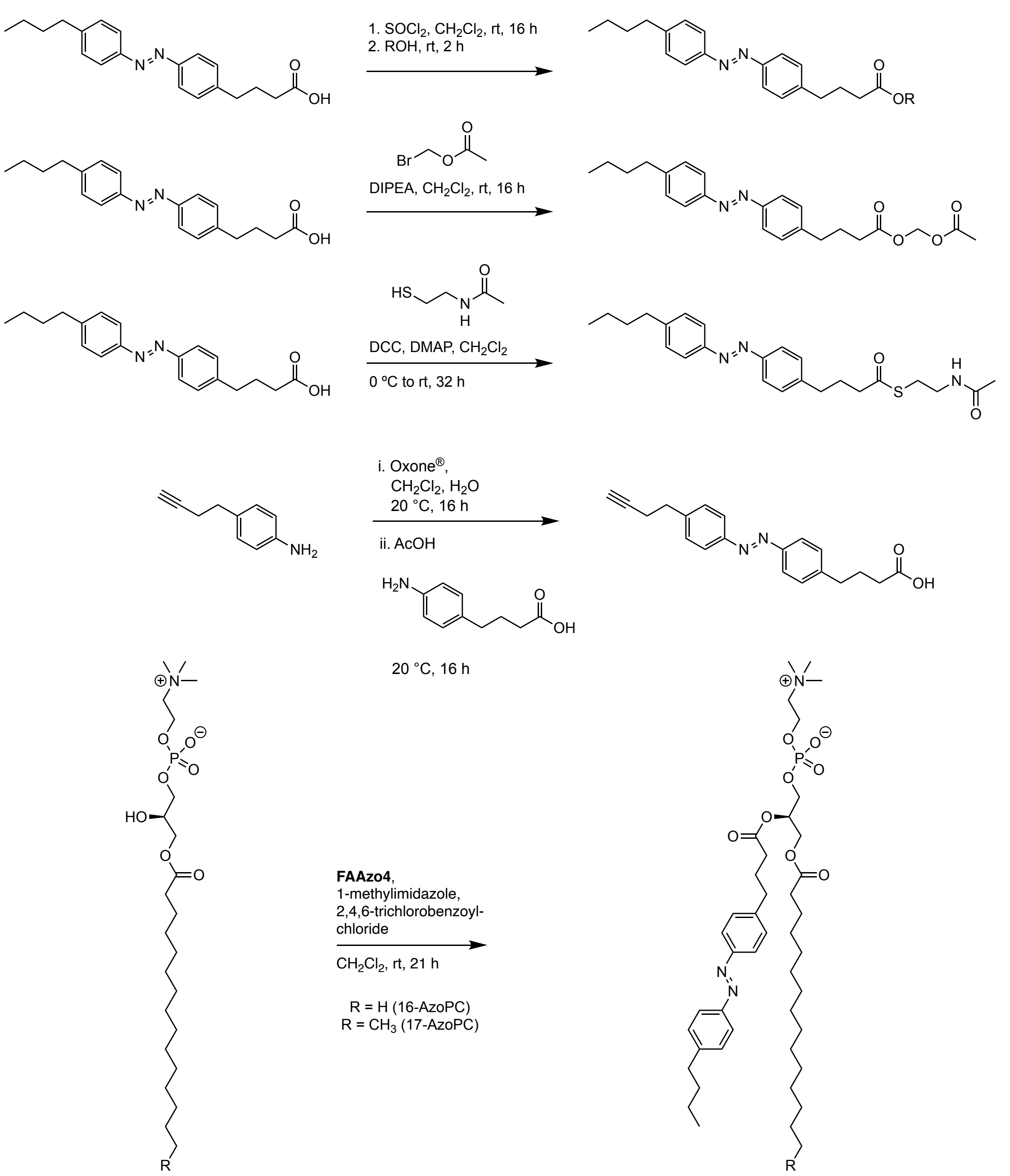

FAAzo4-Me

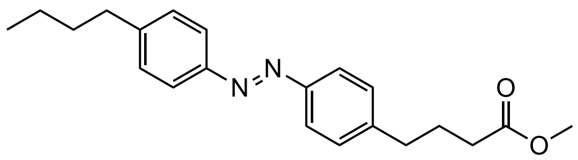

FAAzo4[1] (30.0 mg, 92.5 μmol, 1.00 equiv.) was dissolved in a 1 M $SOCl_2$ (10.0 mL, 10.0 mmol, 108 equiv.) solution in CH_2Cl_2 and stirred at rt for 16 h. Volatiles were removed under reduced pressure, residues were dissolved in 1.3 mL CH_2Cl_2, and MeOH (37.0 μL, 0.925 mmol, 10.0 equiv.) was added. The mixture was stirred at rt for 2 h. The solvent was removed under reduced pressure. The crude was purified using flash column chromatography (hexanes to 30 % EtOAc in hexanes) to yield the product (24.5 mg, 72.4 μmol, 78%) as an orange liquid.

^{1}H NMR (400 MHz, $CDCl_3$) δ 7.83 (dd, *J* = 8.4, 2.3 Hz, 4H), 7.31 (d, *J* = 8.1 Hz, 4H), 3.68 (s, 3H), 2.77 – 2.65 (m, 4H), 2.36 (t, *J* = 7.4 Hz, 2H), 2.01 (p, *J* = 7.5 Hz, 2H), 1.65 (p, *J* = 7.5 Hz, 2H), 1.38 (h, *J* = 7.3 Hz, 2H), 0.94 (t, *J* = 7.3 Hz, 3H).

^{13}C NMR (100 MHz, $CDCl_3$) δ 174.0, 151.4, 151.1, 146.5, 144.7, 129.3, 129.2, 123.0, 122.9, 51.7, 35.7, 35.1, 33.6, 33.5, 26.5, 22.5, 14.1.

HRMS: m/z calc. for $C_{21}H_{27}N_2O_2^+$ ($[M+H]^+$): 339.2067, found: 339.2073.

FAAzo4-Et

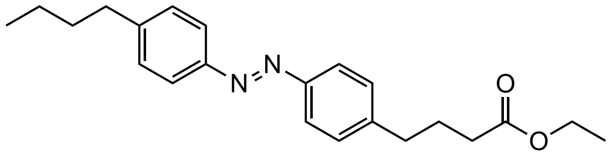

FAAzo4[1] (30.0 mg, 92.5 μmol, 1.00 equiv.) was dissolved in a 1 M $SOCl_2$ (10.0 mL, 10.0 mmol, 108 equiv.) solution in CH_2Cl_2 and stirred at rt for 16 h. Volatiles were removed under reduced pressure, residues were dissolved in 1.3 mL CH_2Cl_2, and EtOH (54.0 μL, 0.925 mmol, 10.0 equiv.) was added. The mixture was stirred at rt for 2 h. The solvent was removed under reduced pressure. The crude was purified using flash column chromatography (hexanes to 30 % EtOAc in hexanes) to yield the product (30.4 mg, 86.2 μmol, 93%) as an orange liquid.

^{1}H NMR (400 MHz, $CDCl_3$) δ 7.83 (dd, *J* = 8.4, 2.3 Hz, 4H), 7.31 (d, *J* = 7.0 Hz, 4H), 4.20 – 4.10 (m, 2H), 2.71 (dt, *J* = 18.3, 7.6 Hz, 4H), 2.35 (t, *J* = 7.4 Hz, 2H), 2.00 (p, *J* = 7.5 Hz, 2H), 1.66 (q, *J* = 7.5 Hz, 2H), 1.38 (h, *J* = 7.3 Hz, 2H), 1.26 (t, *J* = 7.1 Hz, 3H), 0.94 (t, *J* = 7.3 Hz, 3H).

^{13}C NMR (100 MHz, $CDCl_3$) δ 173.5, 151.4, 151.1, 146.5, 144.7, 129.3, 129.2, 123.0, 122.9, 60.5, 35.7, 35.2, 33.7, 33.6, 26.5, 22.5, 14.4, 14.1.

HRMS: m/z calc. for $C_{22}H_{29}N_2O_2^+$ ($[M+H]^+$): 353.2224, found: 353.2228.

FAAzo4-Bu

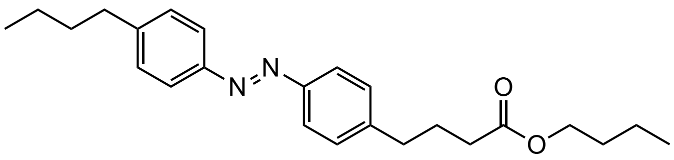

FAAzo4[1] (30.0 mg, 92.5 μmol, 1.00 equiv.) was dissolved in a 1 M $SOCl_2$ (10.0 mL, 10.0 mmol, 108 equiv.) solution in CH_2Cl_2 and stirred at rt for 16 h. Volatiles were removed under reduced pressure, residues were dissolved in 1.3 mL CH_2Cl_2, and BuOH (85.0 μL, 0.925 mmol, 10.0 equiv.) was added. The mixture was stirred at rt for 2 h. The solvent was removed under reduced pressure. The crude was purified using flash column chromatography (hexanes to 30 % EtOAc in hexanes) to yield the product (28.8 mg, 75.7 μmol, 82%) as an orange liquid.

^{1}H NMR (400 MHz, $CDCl_3$) δ 7.83 (dd, *J* = 8.4, 2.4 Hz, 4H), 7.31 (d, *J* = 8.0 Hz, 4H), 4.08 (t, *J* = 6.7 Hz, 2H), 2.71 (dt, *J* = 17.8, 7.6 Hz, 4H), 2.35 (t, *J* = 7.4 Hz, 2H), 2.00 (p, *J* = 7.5 Hz, 2H), 1.63 (tq, *J* = 12.9, 7.0 Hz, 4H), 1.38 (h, *J* = 7.4 Hz, 4H), 0.94 (td, *J* = 7.3, 3.1 Hz, 6H).

^{13}C NMR (100 MHz, $CDCl_3$) δ 173.6, 151.4, 151.1, 146.5, 144.7, 129.3, 129.2, 123.0, 122.9, 64.4, 35.7, 35.2, 33.8, 30.8, 26.5, 22.5, 19.3, 14.1, 13.9.

HRMS: m/z calc. for $C_{24}H_{33}N_2O_2^+$ ($[M+H]^+$): 381.2537, found: 381.2534.

FAAzo4-Am

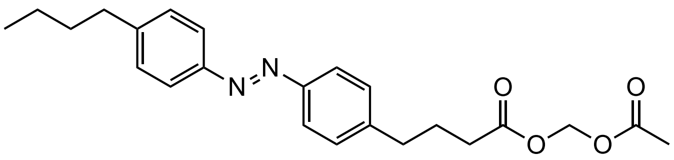

FAAzo4[1] (50.0 mg, 0.154 mmol, 1.00 equiv.) was dissolved in 2 mL CH_2Cl_2, DIPEA (48.0 μL, 0.277 mmol, 1.80 equiv.), and acetoxymethyl bromide (47.2 mg, 0.308 mmol, 2.00 equiv.) were added, and stirred at rt for 16 h. Volatiles were removed under reduced pressure and the crude prduct was purified using flash column chromatography (hexanes to 30 % EtOAc in hexanes) to yield the product (12.5 mg, 31.5 μmol, 20%) as an orange liquid.

^{1}H NMR (400 MHz, $CDCl_3$) δ 7.83 (dd, *J* = 8.3, 2.5 Hz, 4H), 7.31 (dd, *J* = 8.3, 1.6 Hz, 4H), 5.75 (s, 2H), 2.71 (dt, *J* = 20.6, 7.6 Hz, 4H), 2.41 (t, *J* = 7.4 Hz, 2H), 2.03 (q, *J* = 7.5 Hz, 2H), 1.65 (p, *J* = 7.6 Hz, 2H), 1.38 (dq, *J* = 14.7, 7.4 Hz, 2H), 0.94 (t, *J* = 7.3 Hz, 3H).

^{13}C NMR (100 MHz, $CDCl_3$) δ 172.2, 169.8, 151.5, 151.1, 146.5, 144.3, 129.3, 129.2, 123.0, 122.9, 79.3, 35.7, 34.9, 33.6, 33.3, 26.1, 22.5, 20.9, 14.1.

HRMS: m/z calc. for $C_{23}H_{29}N_2O_4^+$ ($[M+H]^+$): 397.2122, found: 397.2123.

FAAzo4-SNAC

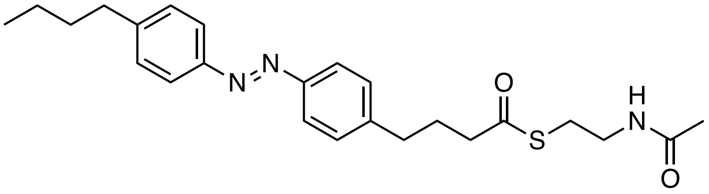

FAAzo4[1] (50.0 mg, 0.154 mmol, 1.00 equiv.) was dissolved in 2 mL CH_2Cl_2 at 0 °C. A solution of DCC (35.0 mg, 0.170 mmol, 1.10 equiv) and DMAP (1.9 mg, 0.015 mmol, 0.10 equiv.) in 1 mL CH_2Cl_2 were added. 2-(acetylamino)ethanethiol (19.3 mg, 0.162 mmol, 1.05 equiv.) was added at 0 °C and stirred at rt for 32 h. Volatiles were removed under reduced pressure and the crude prduct was purified using flash column chromatography (15 % EtOAc in hexanes to 80 % EtOAc in hexanes) to yield the product (60.8 mg, 0.143 mmol, 93%) as an orange solid.

^{1}H NMR (400 MHz, $CDCl_3$) δ 7.82 (dd, *J* = 8.4, 3.4 Hz, 4H), 7.36 – 7.29 (m, 4H), 3.42 (q, *J* = 6.2 Hz, 2H), 3.03 (t, *J* = 6.4 Hz, 2H), 2.77 – 2.66 (m, 4H), 2.61 (t, *J* = 7.4 Hz, 2H), 2.05 (q, *J* = 8.6, 8.1 Hz, 2H), 1.95 (s, 3H), 1.69 – 1.62 (m, 2H), 1.39 (dt, *J* = 14.9, 7.4 Hz, 2H), 0.94 (t, *J* = 7.3 Hz, 3H).

^{13}C NMR (100 MHz, $CDCl_3$) δ 199.7, 170.4, 151.5, 151.1, 146.5, 144.2, 129.3, 129.2, 123.0, 122.9, 43.3, 39.8, 35.7, 34.9, 33.6, 28.7, 27.0, 23.4, 22.5, 14.1.

HRMS: m/z calc. for $C_{24}H_{32}N_3O_2S^+$ ($[M+H]^+$): 426.2210, found: 426.2209.

clFAAzo4

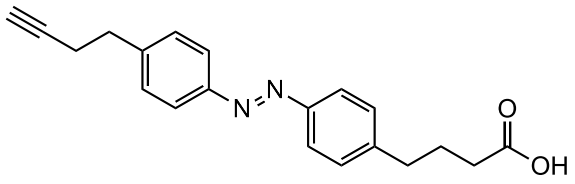

4-(but-3-yn-1-yl)aniline (22.0 mg, 0.123 mmol, 1.00 equiv.) was dissolved in 4 mL CH_2Cl_2. A solution of Oxone® (340 mg, 0.552 mmol, 4.50 equiv.) in 4 mL H_2O was added and stirred vigorously at rt for 16 h. The organic phase was washed with 1 M HCl, a saturated solution of $NaHCO_3$, water, dried over Na_2SO_4, and filtered. 4-(4-aminophenyl)butyric acid (26.7 mg, 0.184 mmol, 1.50 equiv.) and acetic acid (4 mL) were added, CH_2Cl_2 was removed under reduced pressure, and the solution was stirred for 16 h at room temperature. Acetic acid was removed under reduced pressure, azeotroped with toluene, and the product was purified by flash column chromatography (1% AcOH in CH_2Cl_2) to yield **clFAAzo4** (16.5 mg, 51.5 μmol, 42%) as an orange solid.

^{1}H NMR (400 MHz, $CDCl_3$) δ 7.88 – 7.81 (m, 4H), 7.35 (dd, *J* = 18.3, 8.4 Hz, 4H), 2.93 (t, *J* = 7.4 Hz, 2H), 2.75 (h, *J* = 7.4 Hz, 2H), 2.54 (td, *J* = 7.4, 2.6 Hz, 2H), 2.41 (t, *J* = 7.4 Hz, 2H), 2.06 – 1.94 (m, 3H).

^{13}C NMR (100 MHz, $CDCl_3$) δ 178.3, 151.6, 151.4, 144.6, 143.6, 129.3, 129.3, 123.1, 123.0, 83.5, 69.4, 35.0, 34.8, 33.1, 26.2, 20.5.

HRMS: m/z calc. for $C_{20}H_{21}N_2O_2^+$ ($[M+H]^+$): 321.1598, found: 321.1594.

16-AzoPC (16:FAAzo4)

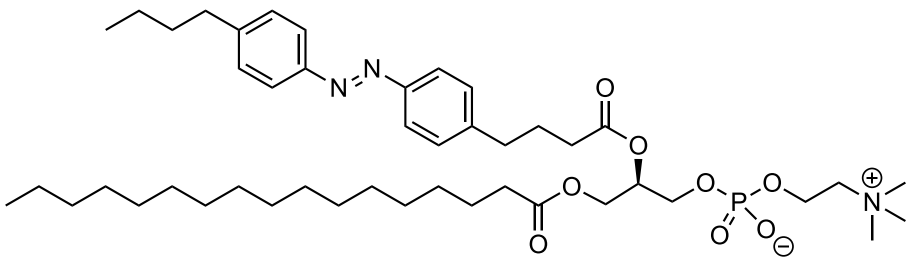

16-LysoPC (50.0 mg, 0.101 mmol, 1.00 equiv.) was dissolved in 30 mL of CH_2Cl_2. 2,4,6-trichlorobenzoyl chloride (73.8 mg, 0.302 mmol, 3.0 equiv.), **FAAzo-4**[1] (65.5 mg, 202 mmol, 2.0 equiv.), and NMI (*N*-methylimidazol) (24.8 mg, 0.302 mmol, 3.0 equiv.) were added to the solution. The clear orange solution was stirred for 16 h at room temperature and was directly subjected to purification via flash column chromatography (CH_2Cl_2, CH_2Cl_2:MeOH:H_2O 99:1:0 / 95:5:0 / 8:2:0,1 / 7.5:2.5:0.1 / 7:3:0,1 / 7:3:0,2 / 10:4:0.5) to yield **16-AzoPC (16:FAAzo4)** (15.6 mg, 19.5 μmol, 19 %) as an orange glue.

^{1}H NMR (400 MHz, MeOD) δ 7.83 (t, *J* = 8.1 Hz, 4H), 7.37 (dd, *J* = 13.1, 8.4 Hz, 4H), 5.32 – 5.22 (m, 1H), 4.50 – 4.41 (m, 1H), 4.28 (d, *J* = 4.5 Hz, 2H), 4.25 – 4.15 (m, 1H), 4.02 (q, *J* = 6.1 Hz, 2H), 3.63 (dd, *J* = 5.6, 3.3 Hz, 2H), 3.21 (s, 9H), 2.74 (dt, *J* = 20.8, 7.7 Hz, 4H), 2.47 – 2.36 (m, 2H), 2.34 – 2.27 (m, 2H), 2.00 (dq, *J* = 11.8, 6.8 Hz, 2H), 1.71 – 1.62 (m, 2H), 1.56 (p, *J* = 7.4 Hz, 2H), 1.42 (dd, *J* = 15.0, 7.5 Hz, 2H), 1.31 – 1.19 (m, 32H), 0.97 (t, *J* = 7.4 Hz, 3H), 0.89 (t, *J* = 6.8 Hz, 4H).

^{13}C NMR (100 MHz, MeOD) δ 175.0, 174.2, 152.5, 152.3, 147.8, 146.4, 130.4, 130.2, 123.9, 123.8, 72.1, 64.9, 63.6, 60.5, 60.5, 54.7, 54.7, 54.6, 49.7, 49.6, 49.5, 49.4, 49.3, 49.2, 49.1, 49.0, 48.8, 48.6, 48.4, 36.5, 35.8, 34.9, 34.8, 34.4, 33.1, 30.8, 30.6, 30.5, 30.4, 30.2, 27.7, 26.0, 23.7, 23.4, 14.5, 14.3.

^{31}P NMR (162 MHz, MeOD) δ -0.6.

17-AzoPC (17:FAAzo4)

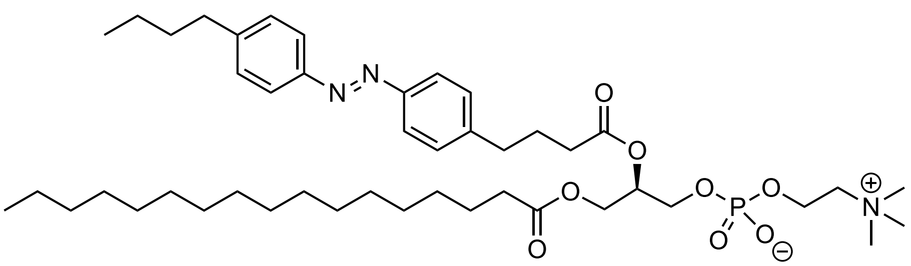

17-LysoPC (10.0 mg, 19.6 μmol, 1.0 equiv.) was dissolved in 620 μL of CH_2Cl_2. 2,4,6-trichlorobenzoyl chloride (14.4 mg, 58.9 μmol, 3.0 equiv.), **FAAzo-4**[1] (12.7 mg, 39.2 μmol, 2.0 equiv.), and NMI (*N*-methylimidazol) (4.8 mg, 58.9 μmol, 3.0 equiv.) were added to the solution. The clear orange solution was stirred for 21 h at room temperature and was directly subjected to purification via flash column chromatography (CH_2Cl_2, CH_2Cl_2:MeOH:H_2O 99:1:0 / 95:5:0 / 8:2:0,1 / 7.5:2.5:0.1 / 7:3:0,1 / 7:3:0,2 / 10:4:0.5) to yield **17-AzoPC (17:FAAzo4)** (15.9 mg, 19.5 μmol, 99 %) as an orange glue.

^{1}H NMR (400 MHz, MeOD) δ 7.83 (t, *J* = 8.0 Hz, 4H), 7.42 – 7.33 (m, 4H), 5.28 (s, 1H), 4.49 – 4.43 (m, 1H), 4.27 (s, 2H), 4.20 (dd, *J* = 12.0, 6.8 Hz, 1H), 4.05 – 3.96 (m, 2H), 3.62 (s, 2H), 3.21 (s, 9H), 2.79 – 2.67 (m, 4H), 2.41 (t, *J* = 7.3 Hz, 2H), 2.30 (t, *J* = 7.5 Hz, 2H), 2.04 – 1.95 (m, 2H), 1.65 (q, *J* = 7.8 Hz, 2H), 1.56 (p, *J* = 7.2 Hz, 2H), 1.41 (dt, *J* = 14.9, 7.4 Hz, 2H), 1.21 (s, 31H), 0.97 (t, *J* = 7.4 Hz, 3H), 0.89 (t, *J* = 6.7 Hz, 3H).

^{13}C NMR (100 MHz, MeOD) δ 173.5, 172.8, 151.1, 150.9, 146.4, 145.0, 129.0, 128.8, 122.5, 122.4, 70.7, 70.6, 66.1, 63.5, 62.2, 59.1, 59.0, 53.3, 53.2, 35.1, 34.4, 33.5, 33.4, 33.0, 31.7, 29.4, 29.4, 29.2, 29.1, 29.0, 28.8, 26.3, 24.6, 22.3, 22.0, 13.1, 12.9.

^{31}P NMR (162 MHz, MeOD) δ -0.6.

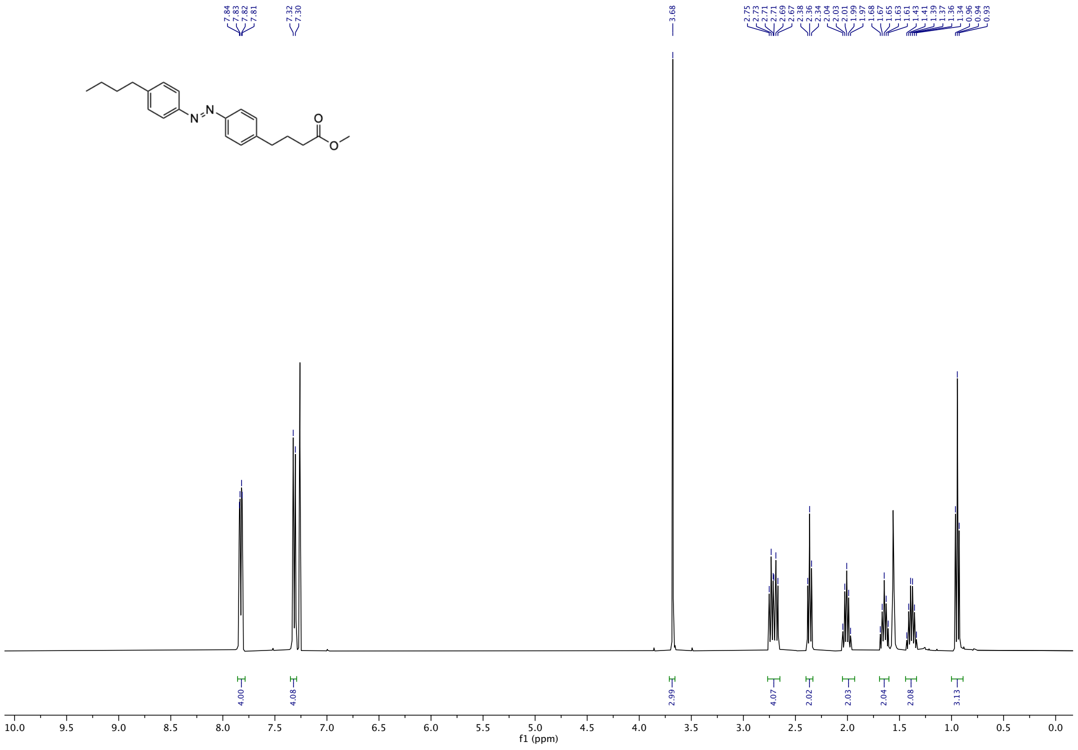
7.84
7.83
7.82
7.81
7.32
7.30
3.68
2.75
2.73
2.71
2.71
2.69
2.67
2.38
2.36
2.34
2.04
2.03
2.01
1.99
1.97
1.68
1.67
1.65
1.63
1.61
1.43
1.41
1.39
1.37
1.36
1.34
0.96
0.94
0.93
4.00
4.08
2.99
4.07
2.02
2.03
2.04
2.08
3.13
f1 (ppm)

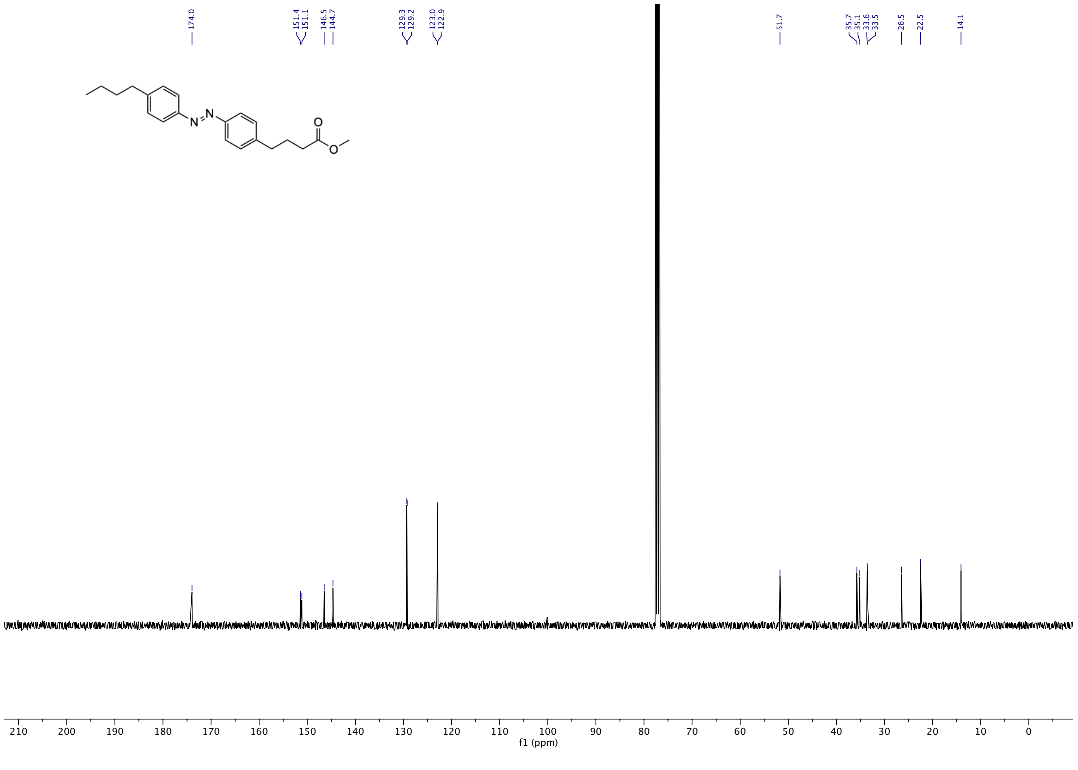
174.0
151.4
151.1
146.5
144.7
129.3
129.2
123.0
122.9
51.7
35.7
35.1
33.6
33.5
26.5
22.5
14.1
f1 (ppm)

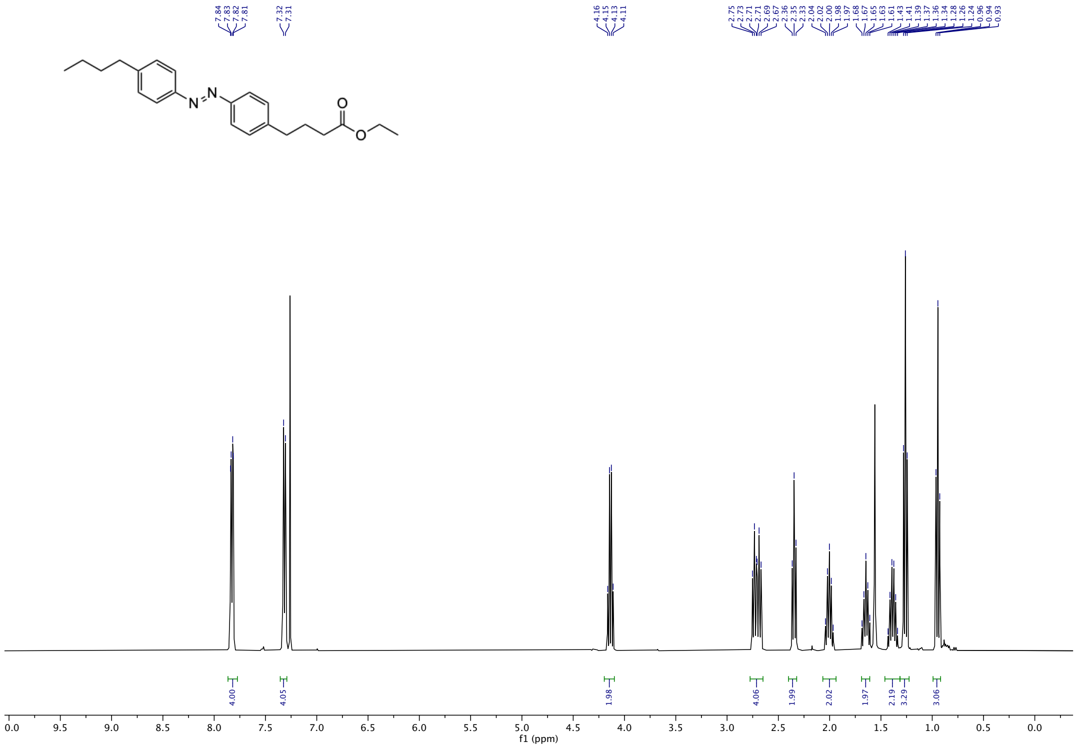
f1 (ppm)

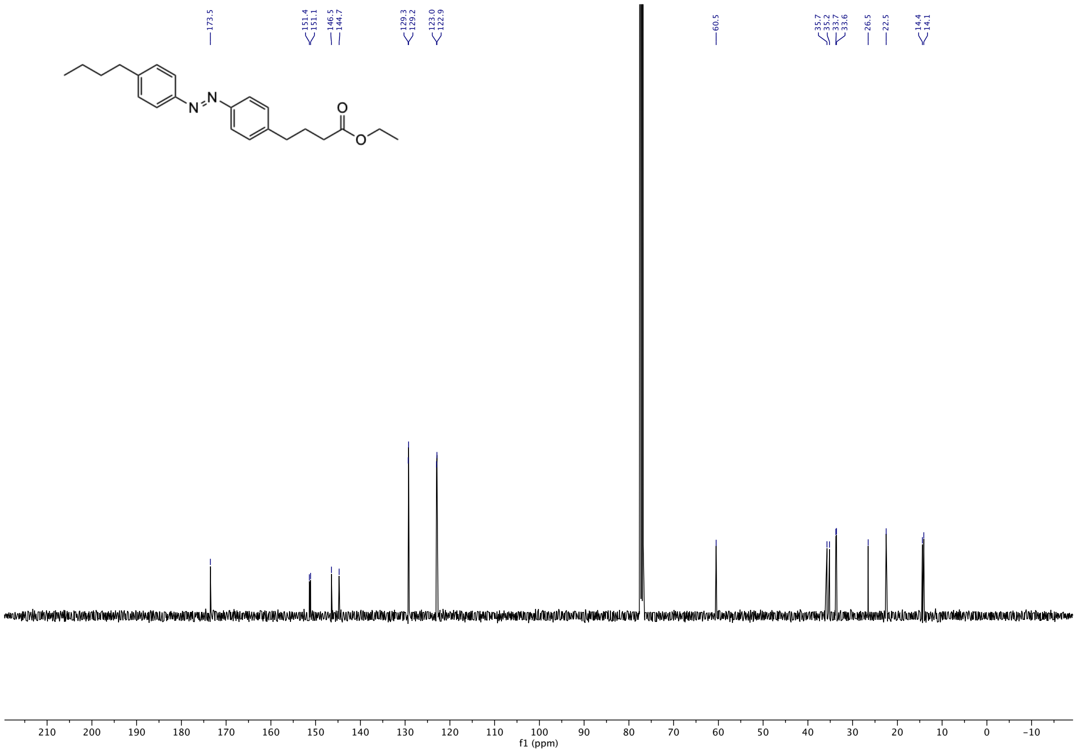
f1 (ppm)

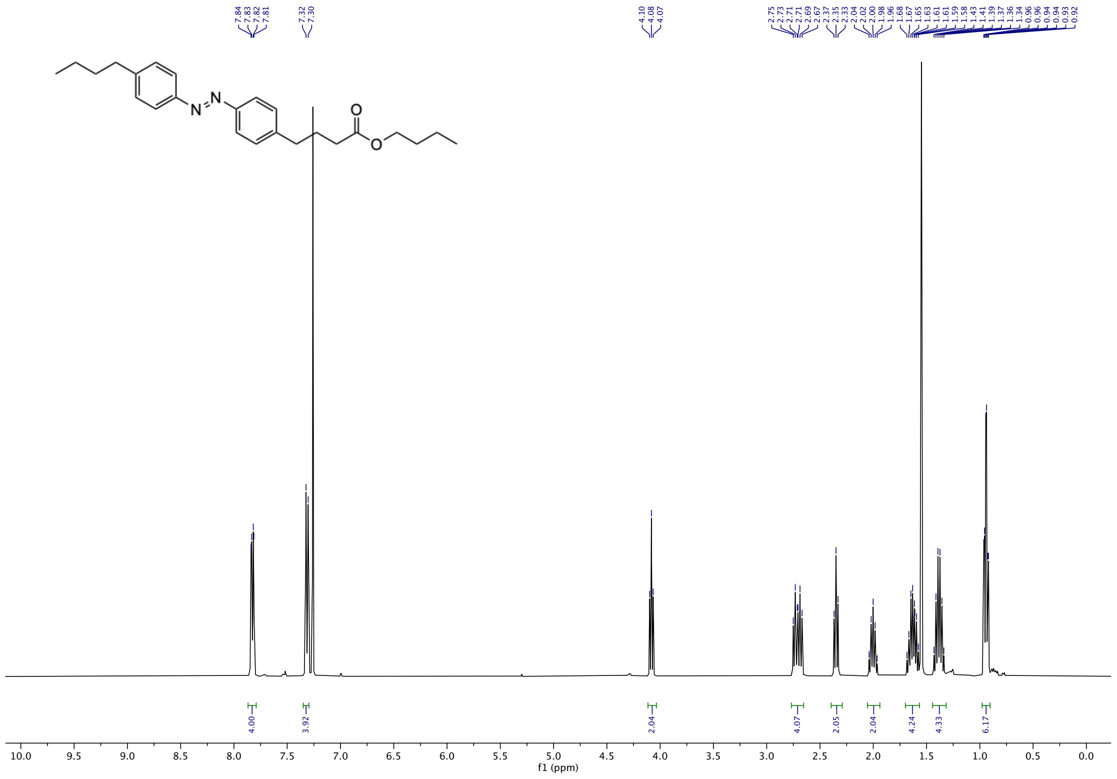

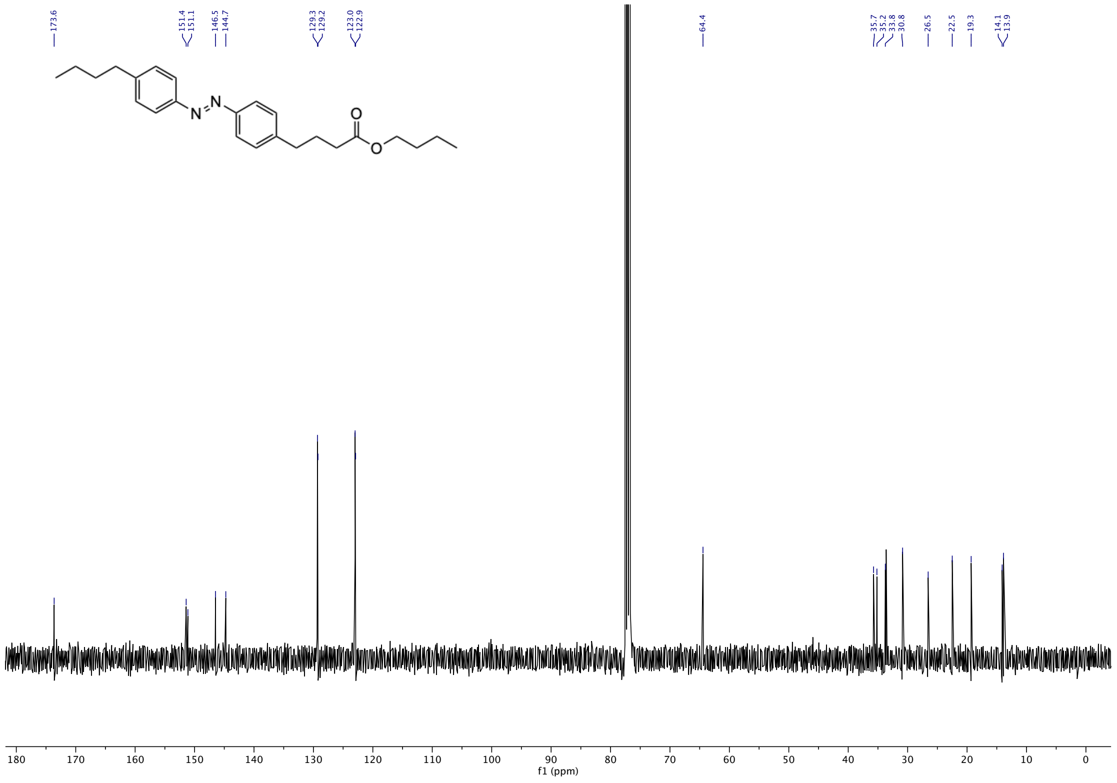

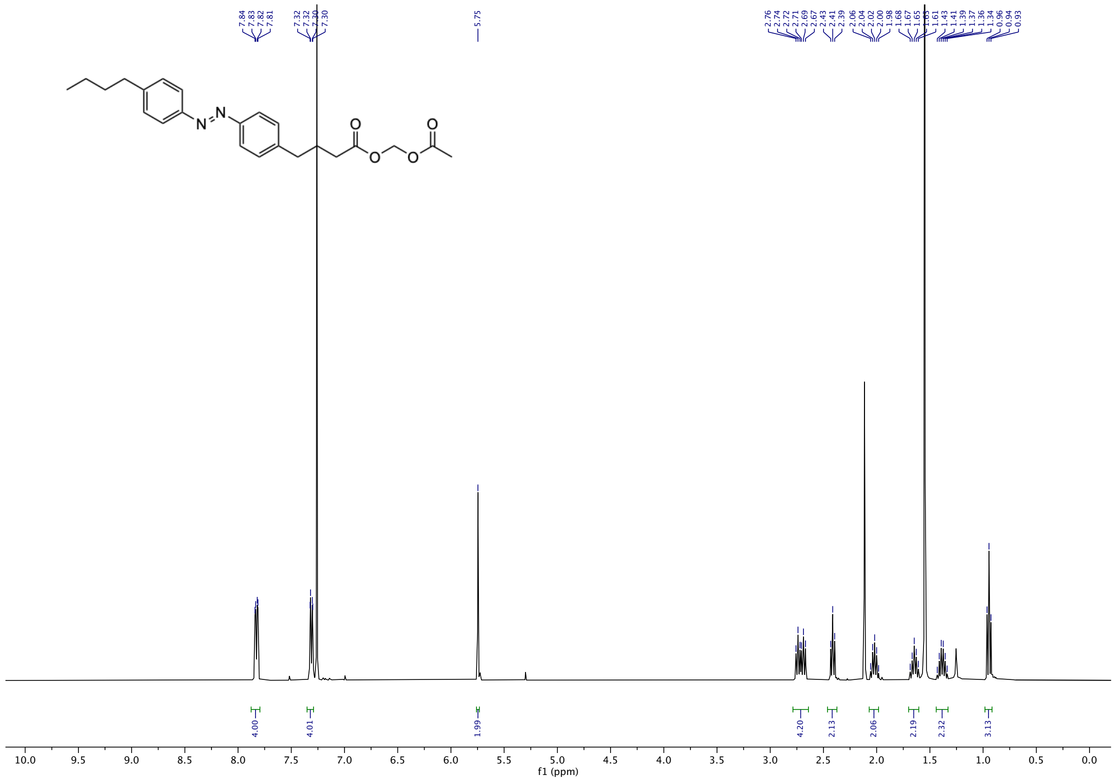
7.84
7.83
7.82
7.81
7.32
7.32
7.30
7.30
5.75
2.76
2.74
2.72
2.71
2.69
2.67
2.43
2.41
2.39
2.06
2.04
2.02
2.00
1.98
1.68
1.67
1.65
1.63
1.61
1.43
1.41
1.39
1.37
1.36
1.34
0.96
0.94
0.93
4.00
4.01
1.99
4.20
2.13
2.06
2.19
2.32
3.13
f1 (ppm)

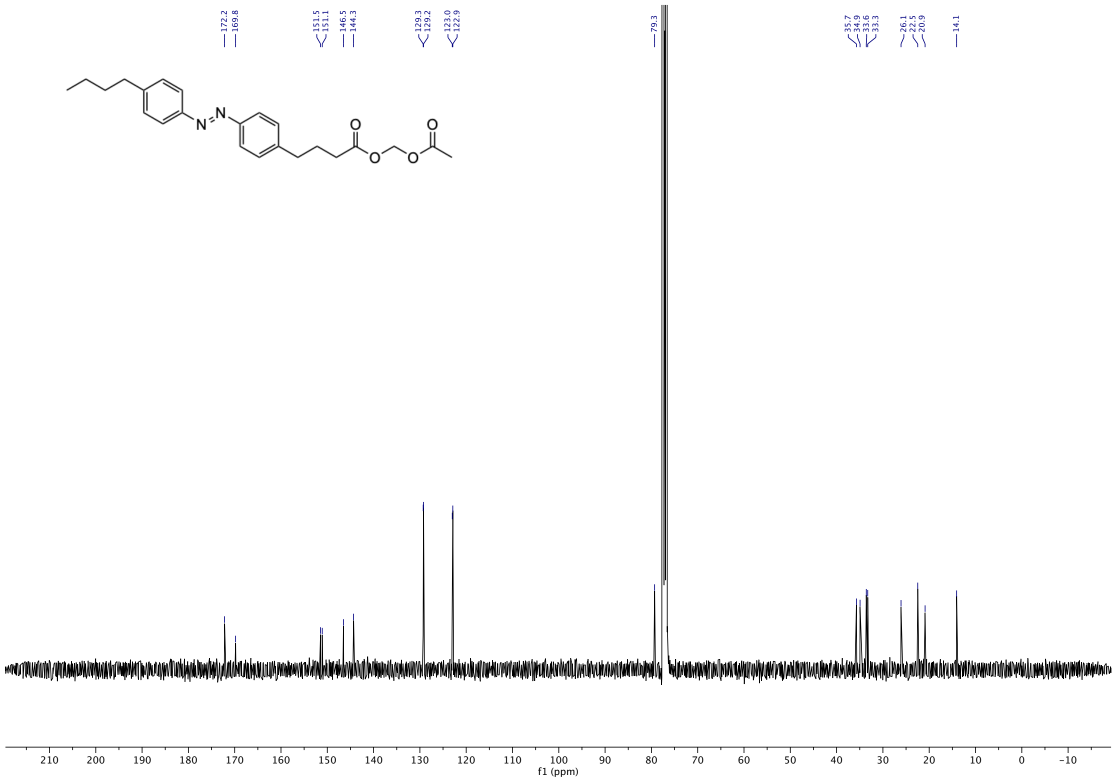
172.2
169.8
151.5
151.1
146.5
144.3
129.3
129.2
123.0
122.9
79.3
35.7
34.9
33.6
33.3
26.1
22.5
20.9
14.1
f1 (ppm)

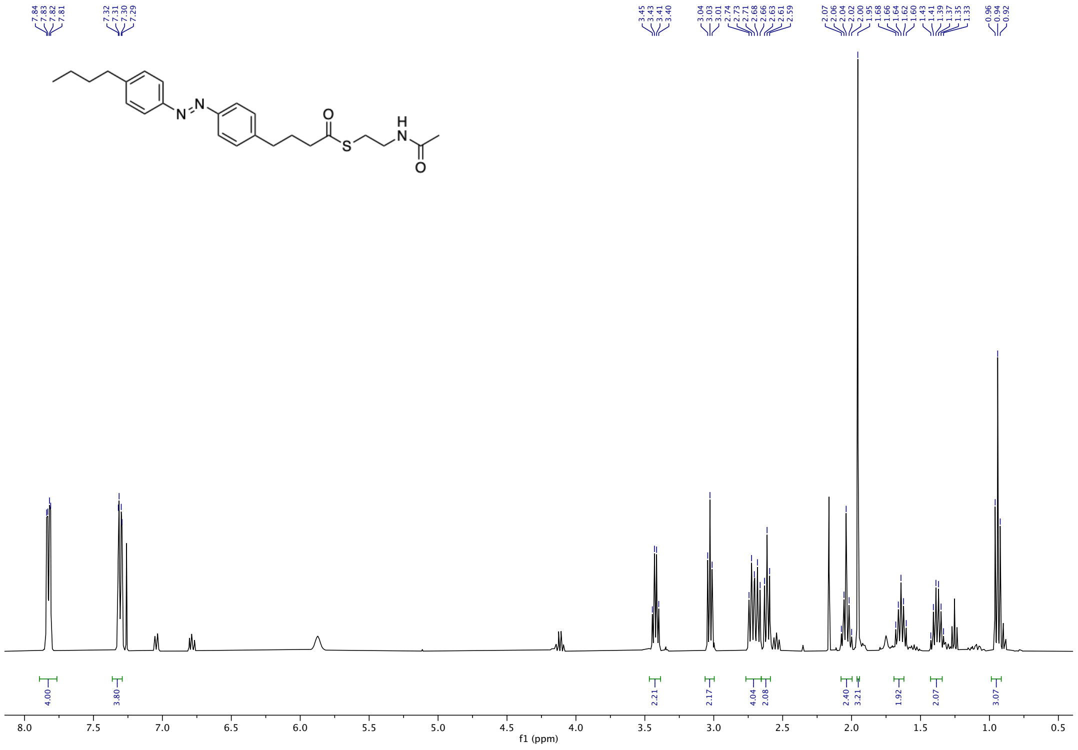

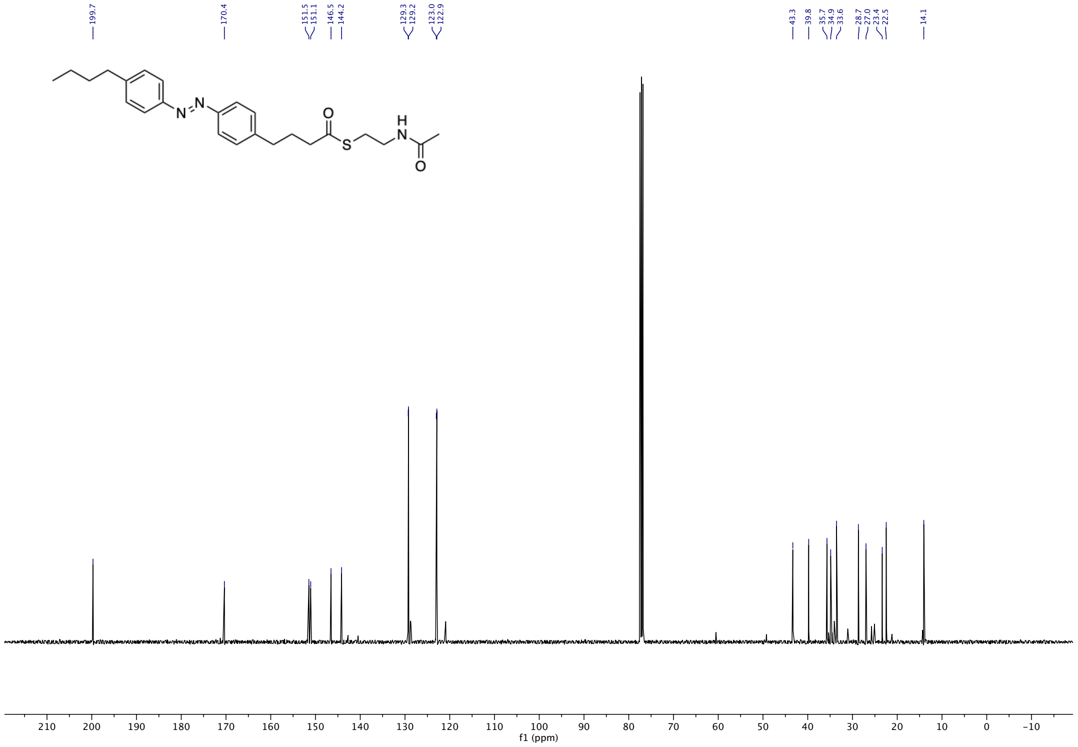

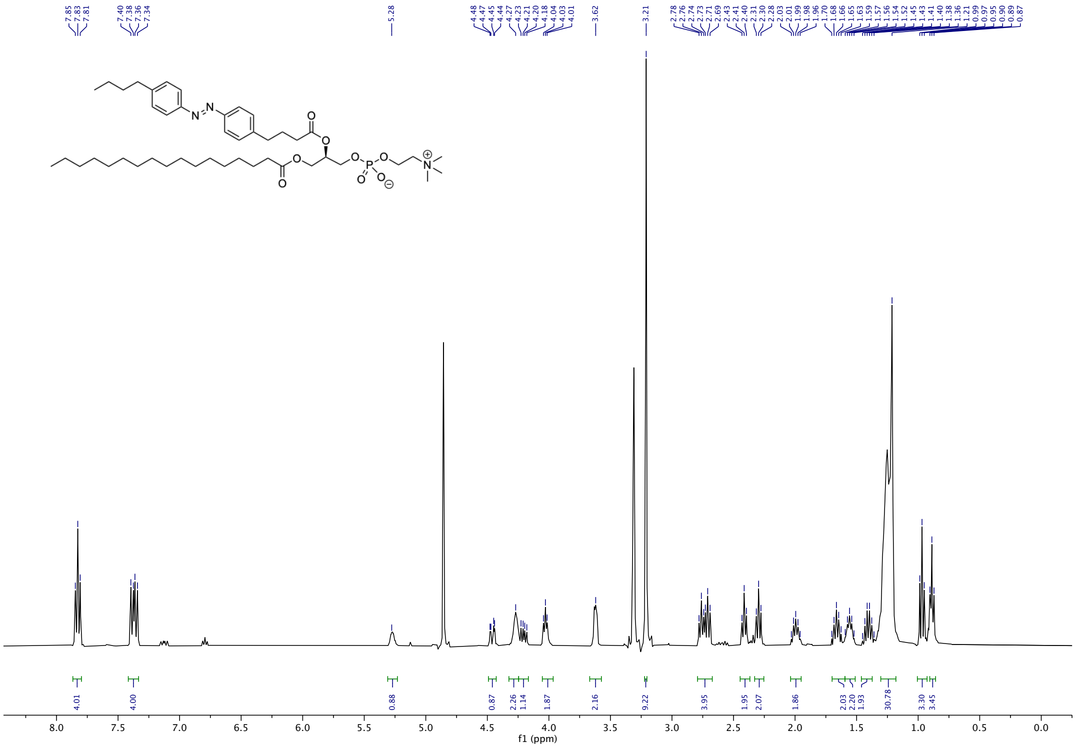
f1 (ppm)

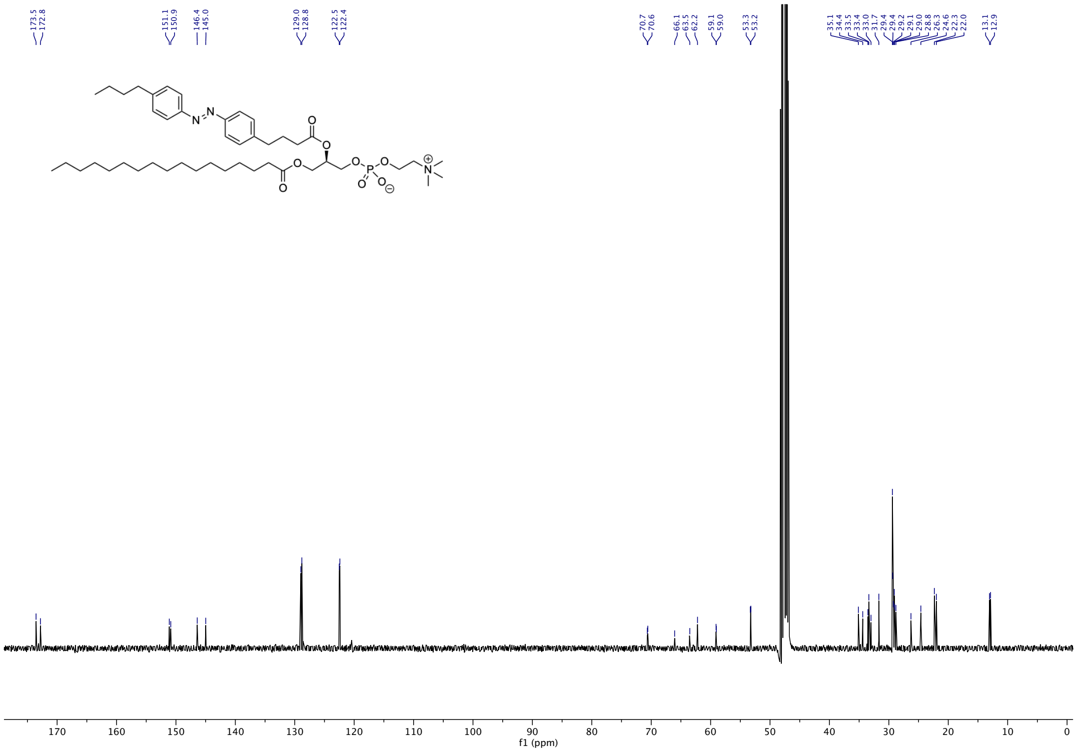
f1 (ppm)

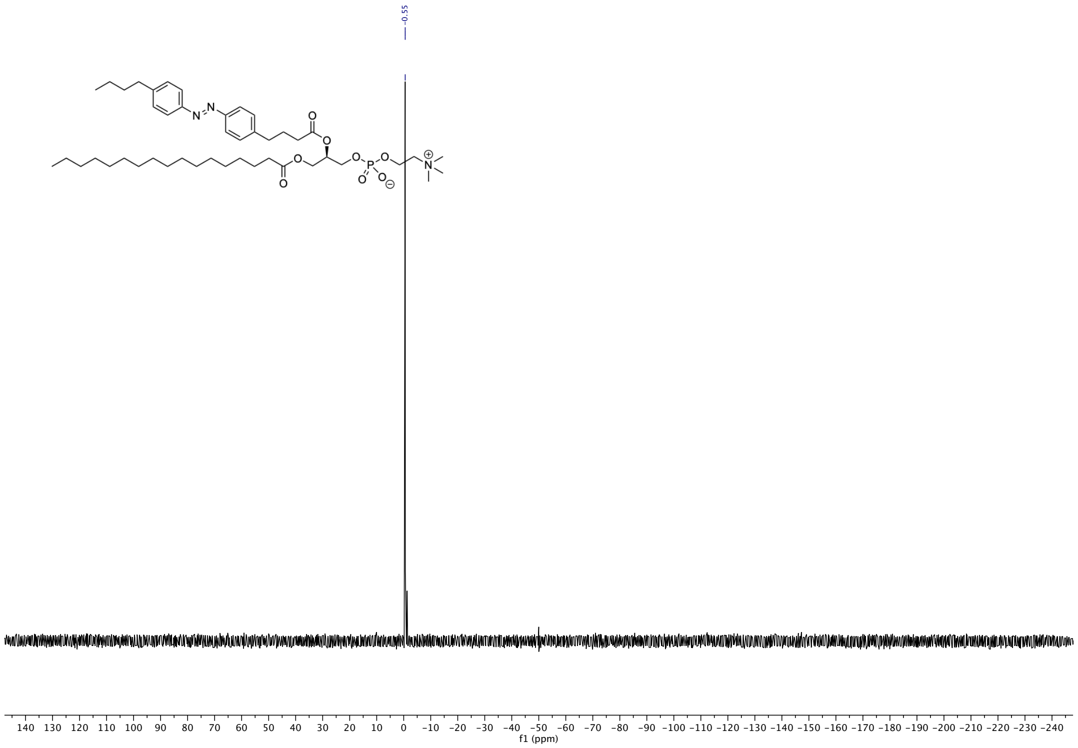
-0.55
f1 (ppm)

References

1. Frank, J.A., Moroni, M., Moshourab, R., Sumser, M., and Lewin, G.R. (2015). Photoswitchable fatty acids enable optical control of TRPV1. *6*, 7118. https://doi.org/10.1038/ncomms8118.
